# Effects of Non-insulin Anti-hyperglycemic Agents on Gut Microbiota: A Systematic Review on Human and Animal Studies

**DOI:** 10.3389/fendo.2020.573891

**Published:** 2020-09-23

**Authors:** Thao T. B. Cao, Kun-Chang Wu, Jye-Lin Hsu, Chih-Shiang Chang, Chiahung Chou, Chen-Yuan Lin, Yu-Min Liao, Pei-Chun Lin, Liang-Yo Yang, Hsiang-Wen Lin

**Affiliations:** ^1^School of Pharmacy and Graduate Institute, China Medical University, Taichung City, Taiwan; ^2^Department of Clinical Pharmacy, Hanoi University of Pharmacy, Hanoi, Vietnam; ^3^Graduate Institute of Biomedical Sciences, China Medical University, Taichung City, Taiwan; ^4^Department of Health Outcomes Research and Policy, Harrison School of Pharmacy, Auburn University, Auburn, AL, United States; ^5^Department of Medical Research, China Medical University Hospital, Taichung City, Taiwan; ^6^Division of Hematology and Oncology, China Medical University Hospital, Taichung City, Taiwan; ^7^Department of Physiology, School of Medicine, College of Medicine, China Medical University, Taichung City, Taiwan; ^8^Laboratory for Neural Repair, China Medical University Hospital, Taichung City, Taiwan; ^9^Biomedical Technology Research and Development Center, China Medical University Hospital, Taichung City, Taiwan; ^10^Department of Pharmacy, China Medical University Hospital, Taichung City, Taiwan; ^11^Department of Pharmacy System, Outcomes and Policy, College of Pharmacy, University of Illinois at Chicago, Chicago, IL, United States

**Keywords:** anti-hyperglycemic drugs, microbiome, microbiota, association, systematic review

## Abstract

**Background:** As growing evidence links gut microbiota with the therapeutic efficacy and side effects of anti-hyperglycemic drugs, this article aims to provide a systematic review of the reciprocal interactions between anti-hyperglycemic drugs and gut microbiota taxa, which underlie the effect of the gut microbiome on diabetic control via bug-host interactions.

**Method:** We followed the PRISMA requirements to perform a systematic review on human vs. animal gut microbiota data in PubMed, SCOPUS, and EMBASE databases, and used Cochrane, ROBIN-I, and SYRCLE tools to assess potential bias risks. The outcomes of assessment were trends on gut microbiota taxa, diversity, and associations with metabolic control (e.g., glucose, lipid) following anti-hyperglycemic treatment.

**Results:** Of 2,804 citations, 64 studies (17/humans; 47/mice) were included. In human studies, seven were randomized trials using metformin or acarbose in obese, pre-diabetes, and type 2 diabetes (T2D) patients. Treatment of pre-diabetes and newly diagnosed T2D patients with metformin or acarbose was associated with decreases in genus of *Bacteroides*, accompanied by increases in both *Bifidobacterium* and *Lactobacillus*. Additionally, T2D patients receiving metformin showed increases in various taxa of the order *Enterobacteriales* and the species *Akkermansia muciniphila*. Of seven studies with significant differences in beta-diversity, the incremental specific taxa were associated with the improvement of glucose and lipid profiles. In mice, the effects of metformin on *A. muciniphila* were similar, but an inverse association with *Bacteroides* was reported. Animal studies on other anti-hyperglycemic drugs, however, showed substantial variations in results.

**Conclusions:** The changes in specific taxa and β-diversity of gut microbiota were associated with metformin and acarbose in humans while pertinent information for other anti-hyperglycemic drugs could only be obtained in rodent studies. Further human studies on anti-hyperglycemic drugs other than metformin and acarbose are needed to explore gut microbiota's role in their therapeutic efficacies and side effects.

## Introduction

Gut microbiota plays a pivotal role in the pathogenesis of diabetes as significant alterations were found in the gut microbiome composition in type 2 diabetes (T2D) patients relative to healthy individuals (1). A metagenome-wide association study reported a moderate degree of dysbiosis associated with depletion in butyrate-producing bacteria, accompanied by increases in opportunistic pathogens among diabetic patients (2). These changes were echoed by a recent systematic review, which shows an inverse association of T2D with the genera *Bifidobacterium, Akkermansia* and butyrate-producing bacteria (e.g., *Roseburia, Faecalibacterium*), in conjunction with a positive association with *Ruminococcus, Fusobacterium*, and *Blautia* (1).

From a clinical perspective, these findings provide a rationale for targeting gut microbiota imbalance as a potential strategy for T2D treatment by restoring a healthy gut microbiome, including fecal microbiota transplant and probiotic supplements (3, 4). However, the efficiency and effectiveness of these treatments remain uncertain due to concerns over the invasive nature of fecal microbiota transplant and the dosage, species, and duration required for an effective probiotic treatment. Emerging evidence indicates that the therapeutic efficacy of anti-hyperglycemic drugs might, in part, be attributable to their ability to modulate the compositions of gut microbiota (1, 3, 5–9). This compositional change might lead to enrichments in bacterial species exhibiting beneficial effects to intestinal health *via* the production of health-promoting metabolites, such as short-chain fatty acids (SCFAs) and bile acids (8). Nevertheless, certain anti-hyperglycemic drugs were reported to cause increases in the abundance of *Escherichia* and *Candidatus Arthromitus*, which contribute to gastrointestinal side effects and weight gain, respectively (9–11).

Among various anti-hyperglycemic drugs in clinical use, metformin, acarbose, sitagliptin, and vildagliptin (5, 6) have been investigated for their reciprocal interplay with gut microbiota by assessing their effects on human and animal gut microbiota, and *vice versa* (1, 8, 11). From a translational perspective, animal models might help to explore the causality of complex host-microbiota interactions and possible mechanisms of action in a controlled experimental setting. However, it should be noted that differences in dietary habits, host metabolism, inflammatory states, and body anatomy contribute to great variations in gut microbiota compositions between humans and animals, and subsequently, the respective drug effects in disease control (12). A meta-analysis of published 16S rDNA sequencing data from mouse and human fecal microbiota showed that there were significant increases in *Lactobacillus* and *Turicibacter* genera in mouse gut microbiota while the genera of *Streptococcus, Ruminococcus, Lachnospira, Faecalibacterium, Dialister*, and *Oscillospira* were elevated in human gut microbiota (12). Moreover, age, mouse strains/populations, microbiota pools in laboratories, and other practical factors might have varied to a great extent among different studies (12). Previous reviews have suggested the effects of anti-hyperglycemic drugs on gut microbiota (8, 11, 13), however, the differences in results between human and animal studies have not been differentiated.

Reciprocal interplays between individual anti-hyperglycemic drugs and gut microbiota remain unexplored with respect to the contribution of specific bacterial taxa to drug's therapeutic efficacy in disease control (i.e., the clinical question). Thus, we conducted this systematic review aiming to shed light on the associations among anti-hyperglycemic agents, changes in specific taxonomic groups of gut microbiota, and host glucose control or metabolic profiles mainly in humans, as compared to those reported in animal studies.

## Methods

### Literature Search

Our literature search strategies were designed to integrate the following PICOS (population, intervention, comparisons, outcomes, study design) based on the prior clinical question: Population: humans (e.g., healthy people or patients who were either obese, prediabetes, diabetes) or the corresponding animal models; Intervention: non-insulin anti-hyperglycemic drugs; Comparisons: post- vs. pre-intervention, with-vs. without-treatment, or on-vs. off-treatment; Outcomes: alteration of the gut microbial composition; Study design: clinical trials, observational studies or animal experiments, as that recommended for systematic reviews (14). We systematically searched PubMed, EMBASE, and SCOPUS databases from January 1, 2000, to November 13, 2019. The keywords and searching strategies based upon the PICOS were “anti-hyperglycemic drugs” and “gut microbiota” related terms (Supplementary Table 1). In addition, we searched manually the reference list of the review papers for additional publications of interest.

### Study Selection Criteria

We followed the preferred reporting items for systematic reviews and meta-analysis (PRISMA) guidelines (15). The inclusion criteria for the published studies included: (i) any human studies or animal experiments reporting original data of gut microbiota after receiving anti-hyperglycemic drugs; (ii) gut microbiota data were analyzed from feces or colonic content specimens; (iii) must be written in English or Chinese. Studies were excluded if they did not provide data of individual bacterial taxa or were only available as conference abstracts or proceedings.

### Selection of Studies

Initially, the abstracts and titles of potential articles were screened, followed by the evaluation of the full-text articles for eligibility. Two authors were responsible for screening and evaluating these papers independently. Disagreements were resolved by consensus between these two authors and, if necessary, discussed by additional two authors.

### Data Extraction

A standardized form in a Microsoft Excel file (e.g., characteristics of studies, participants, treatments and comparisons, methods to analyze the microbiome, and measures of outcomes) was used for data extraction. Data were extracted by one author and reviewed by a second one. All disagreements were resolved by consensus and a third or fourth author when necessary.

### Quality Assessment

We used the Cochrane risk-of-bias tool to assess the risk of bias in selected randomized trials (16). For quasi-experimental and observational studies, we used the Risk of Bias in Non-randomized studies-of Interventions (ROBIN-I) to assess the risk of bias (17). Further, the SYRCLE's risk of bias tool for animal studies (18) was used to assess the risk of bias. The risks of bias data were extracted by four different authors and all disagreements were resolved by consensus made by the remaining authors.

### Outcomes of Assessment

Other than describing the characteristics of the evaluated human or animal studies, the primary outcome was the difference in relative abundance or change patterns of individual intestinal bacterial taxa, categorized based on six common taxonomic categories [Phylum (P)c, Class (C), Order (O), Family (F), Genus (G), Species (S)], in associations with the use of anti-hyperglycemic agents, among those available human or animal studies, respectively. Secondary outcomes were differences in microbial diversity, changes in intestinal or serum levels of SCFAs and/or bile acids in human or animal hosts, respectively, after taking/using individual drugs, associations between specific taxa and host metabolic parameters, e.g., glucose, body weight, and lipid profile.

### Data Synthesis

We classified the primary and secondary outcomes into the following categories: significant increase, significant decrease, and no significant difference between comparison groups. Changes of each taxon were synthesized from at least 2 studies for human or animal studies, respectively. Specifically, the effects of different anti-hyperglycemic drugs on specific taxon among the evaluated human or animal studies were compared. Further, the corresponding effects of each individual drug on specific taxon were compared to explore its consistency, in terms of having the same trend of alteration on the specific taxon caused by the same specific anti-hyperglycemic drug, or not. These findings also were categorized by the target research populations (e.g., obese, pre-diabetic, newly T2D, prevalent T2D), individual treated drugs or different animal models (mice or rat models with various diets or genetic knockout). For gut microbial diversity, each study might use one or more measures to assess α- (richness and evenness) or β-diversity. We considered α-diversity as “Increase” if at least one measure showed an increase and no measure showed a decrease; “Decrease” if at least one measure showed a decrease and no measure showed an increase; “No difference” if all measures showed no difference. β-diversity was assessed as “Difference” if at least one measure showed a difference; “No difference” if all measures showed no difference. In terms of associations between specific taxa and host metabolic parameters, we collected data from specific taxa that increased or decreased significantly in participants receiving non-insulin anti-hyperglycemic drugs. Only data with statistical significance were extracted for analyses.

## Results

The following presented results were mainly focused on human studies, which are compared to those of animal studies.

### Reviewed Studies

**Figure 1:**
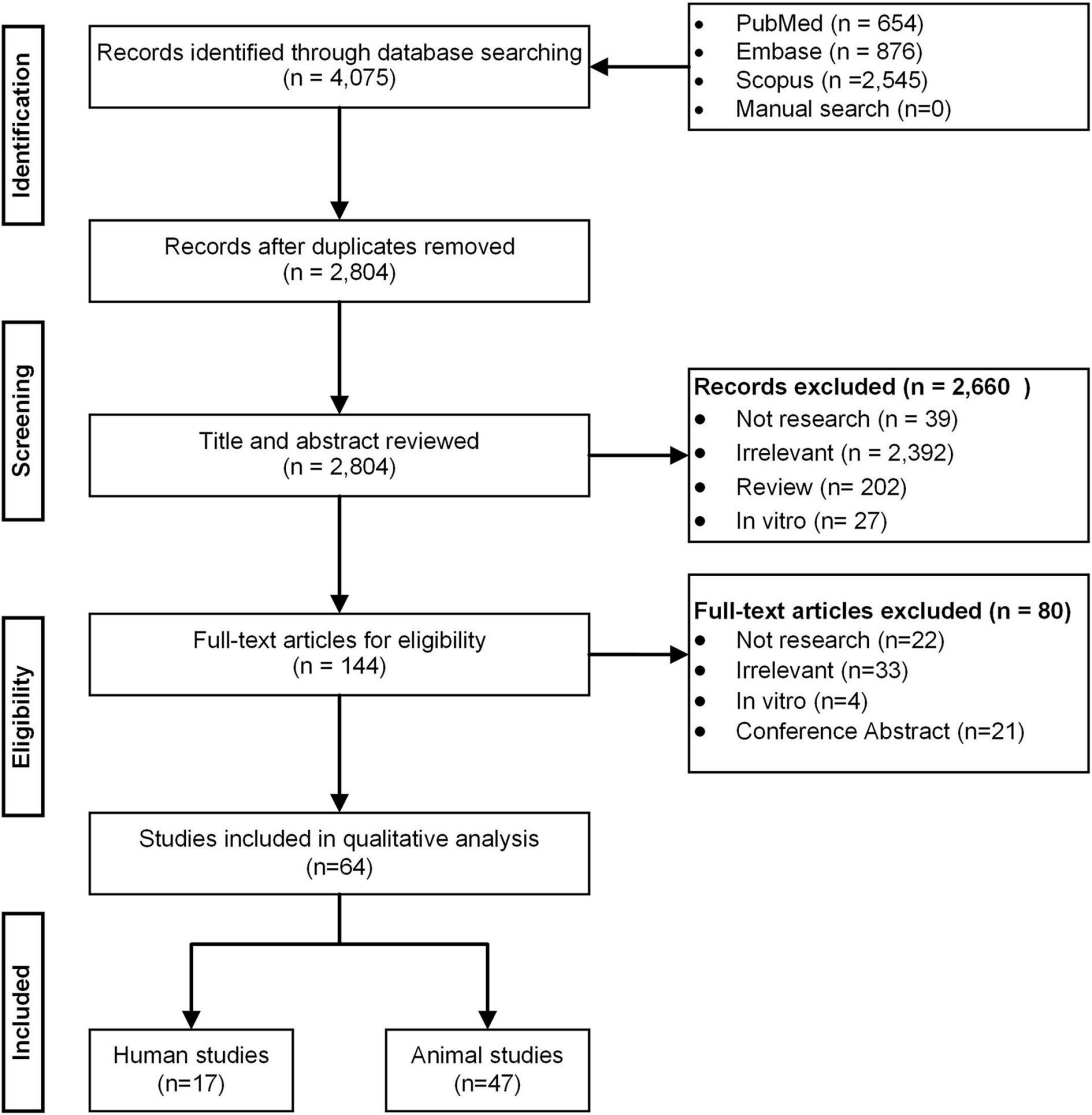
PRISMA flow diagram.

Overall, 2,804 citations were retrieved, and the final analysis included 17 human studies (7, 19–34) and 47 mouse studies (5, 6, 35–79) from 64 papers (Figure 1). The majority of studies were published in and after 2017 and the duration of anti-hyperglycemic treatments varied from studies to studies (i.e., from few days to several months). Of 17 human studies, seven (41.2%) were randomized control trials (7, 19–21, 32–34). Thirteen studies (76.4%) enrolled either newly diagnosed or prevalent T2D patients (20, 21, 23, 24, 26–34), whereas the remaining four studies enlisted healthy participants (22, 25), obese individuals (19), and pre-diabetic patients (7) (Tables 1, 2).

**Table 1:** General characteristics of all included studies.

**Characteristics**		**Human studies (*****N*** **=** **17)**		**Animal studies (*****N*** **=** **47)**	
		***n***	**%**	***n***	**%**
**Study design**	Randomized trials	7	41.2	–	–
	Quasi-experimental studies	5	29.4	–	–
	Cross-sectional studies	5	29.4	–	–
	Animal experiments	–	–	47	100
**Treatment**					
Biguanide	Metformin	14	82.4	24	51.1
α-glucosidase inhibitors	Acarbose	3	17.6	6	12.8
	Voglibose	–	–	2	4.3
	Miglitol	–	–	1	2.1
GLP-1 receptor agonists	Liraglutide	1	5.9	7	14.9
DPP-4 inhibitors	Sitagliptin	–	–	4	8.5
	Vildagliptin	–	–	2	4.3
	Saxagliptin	–	–	1	2.1
	Anagliptin	–	–	1	2.1
SGLT-2 inhibitors	Dapagliflozin	–	–	2	4.3
	Canagliflozin	–	–	1	2.1
Thiazolidindiones	Pioglitazone	–	–	1	2.1
	Rosiglitazone	–	–	1	2.1
Sulfonylure	Glipizide	1	5.9	–	–
**Published year**	2019	3	17.6	14	29.8
	2018	7	41.2	16	34.0
	2017	4	23.5	6	12.8
	2016	–	–	5	10.6
	2011–2015	3	17.6	6	12.8
**Geography**	Asia	7	41.2	33	70.2
	Europe	7	41.2	5	10.6
	North America	2	11.7	8	17.0
	South America	1	5.9	1	2.1
**Participants**					
Human	Newly T2D	4	23.5	–	–
	Prevalent T2D	9	52.9	–	–
	Healthy	2	11.8	–	–
	Obese	1	5.9	–	–
	Pre-diabetic	1	5.9	–	–
Mice/rats	Diet or STZ or both	–	–	29	61.7
	Gene knockout	–	–	12	25.5
	Diet and gene knockout	–	–	2	4.3
	Wild type with normal diet	–	–	6	12.7
	Other (adenine)	–	–	1	2.1
**Specimens**	Feces	17	100	34	72.3
	Intestinal, colon, cecal contents	–	–	11	23.4
	Feces and intestinal, colon contents	–	–	2	4.3
**Assessment methods**	T-RFLP	1	5.9	–	–
	RT-qPCR	1	5.9	6	12.8
	Metagenomic sequencing	5	29.4	–	–
	16S rRNA gene sequencing	10	58.8	35	74.4
	16S rRNA gene sequencing and metagenomic	–	–	1	2.1
	16S rDNA gene sequencing	–	–	3	6.4
	DGGE	–	–	1	2.1
	Cultivation	–	–	1	2.1
**Variable gene region for gene sequencing**	V3–V4	2	11.8	15	31.9
	V4	3	17.6	9	19.1
	V3	1	5.9	4	8.5
	V1–V2	1	5.9	3	6.4
	V1–V3	–	–	5	10.6
	V1, V2, V3	1	5.9	–	–
	V3–V5	1	5.9	–	–
	V4–V5	–	–	1	2.1
	V5–V6	–	–	1	2.1
	Not stated	1	5.9	1	2.1

*T-RFLP, terminal restriction fragment length polymorphism; RT-qPCR, Real-time quantitative polymerase chain reaction; DGGE, denaturing gradient gel electrophoresis; –, no information*.

**Table 2:** Characteristics of included human studies.

**References, country**	**Participants**	***N***	**Treatment and daily dose**	**Duration**	**Specimen**	**Analysis method**	**Comparison**	**Outcomes**				
								**α-diversity**	**β- diversity**	**Taxonomic composition**	**SCFAs**	**Bile acids**
**Randomized trials (*****n*** **=** **7)**												
Ejtahed et al. (19), Iran	Obese	20/16	M (1,000 mg) vs. placebo	2 m	Feces	16S rRNA	Post vs. pre	√	√	√	√	
Tong et al. (20), China	Newly T2D	100/100	Chinese medicine vs. M (750 mg)	12 w	Feces	16S rRNA V3–V4 region	Post vs. pre	√	√	√		
Wu et al. (21), Europe	Newly T2D	22/18	M (425–1,700 mg) vs. placebo	4 m	Feces	DNA shotgun metagenomics	Post vs. pre		√	√		√
							With vs. without				√	√
Zhang et al. (7), China	Pre-diabetes	40/40	A (50–150 mg) vs. placebo	4 w	Feces	16S rRNA V3–V5 region	Post vs. pre	√	√	√		
Gu et al. (32), China	Newly T2D	51/43	A (75–450 mg) vs. G (5–15 mg)	3 m	Feces	DNA metagenomics	Post vs. pre	√		√		√
Su et al. (33), China	Prevalent T2D	59/36	A 150 mg vs. non-A	4 w	Feces	16S rDNA RT-qPCR	Post vs. pre			√		
Wang et al. (34), USA	Prevalent T2D	19/18	L vs. M (as usual)	18 w	Feces	16S rRNA V4 region	L vs. M	√	√	√		
							Post vs. pre		√	√		
**Quasi-experimental studies (*****n*** **=** **5)**												
Bryrup et al. (22), Denmark	Healthy	25	M (500–2,000 mg)	6 w	Feces	16S rRNA V4 region	Post vs. pre	√	√	√		
Huang et al. (23), Sweden	Prevalent T2D	23/7	M (as usual) vs. non-M	28 w	Feces	16S rRNA gene T-RFLP	With vs. without	√		√	√	
Sun et al. (24), China	Newly T2D	22	M (2,000 mg)	3 d	Feces	DNA metagenomics	Post vs. pre	√	√	√		√
Elbere et al. (25), Latvia	Healthy	18	M (1,700 mg)	7 d	Feces	16S rRNA V3 region	Post vs. pre	√	√	√		
Napolitano et al. (26), UK	Prevalent T2D	14/14	on-M (as usual) vs. off-M	NA	Feces	16S rRNA V1, V2, V3 regions	On vs. off		√	√		√
**Cross-sectional studies (*****n*** **=** **5)**												
Zhang et al. (27), China	Prevalent T2D	51/26	M (as usual) vs. non-treatment	–	Feces	16S rRNA V1–V2 region	With vs. without	√	√	√		
Barengolts et al. (28), USA	Prevalent T2D	25/16	M (as usual) vs. non-M	–	Feces	16S rRNA V3–V4 region	With vs. without	√	√	√		
De La Cuesta-Zuluaga et al. (29), Columbia	Prevalent T2D	14/14	M (as usual) vs. non-M	–	Feces	16S rRNA V4 region	With vs. without	√	√	√		
Forslund et al. (30), Denmark	Prevalent T2D	58/17	M (as usual) vs. non-M	–	Feces	16S rDNA shotgun metagenomics	With vs. without	√		√		
Karlsson et al. (31), Sweden	Prevalent T2D	20/33	M (as usual) vs. non-M	–	Feces	DNA metagenomics	With vs. without			√		

*A, acarbose; G, glipizide; M, metformin; L, liraglutide; T-RFLP, terminal restriction fragment length polymorphism; RT-qPCR, Real-time quantitative polymerase chain reaction; m, months; w, weeks; d, days; –, no information; NA, not available; vs., versus*.

Of 47 rodent studies, 30 (63.8%) studies were conducted in mice (35–41, 44–46, 48, 52, 53, 55, 56, 59–65, 68, 70, 74–79) and the others were in rats (5, 6, 42, 43, 47, 49–51, 54, 57, 58, 66, 67, 69, 71–73). Their characteristics, housing, acclimatization, and diet treatments were presented in Table 3 and Supplementary Table 2. Overall, 14 anti-hyperglycemic agents were used in these included studies, with metformin accounting for the most. There was only one human study focused on glipizide, while the other ten listed drugs (e.g., voglibose, miglitol, vildagliptin, sitagliptin, saxagliptin) were used only in rodent studies (Table 1).

**Table 3:** Characteristics of included animal studies.

**References, country**	**Animals**	**Models**	***N***	**Treatment and daily dose**	**Duration**	**Specimens**	**Analysis methods**	**Comparison**	**Outcomes**				
									**α-diversity**	**β-diversity**	**Taxonomic composition**	**SCFAs**	**Bile acids**
**Studies in mice (*****n*** **=** **30)**													
Ryan et al. (37), Ireland	Male C57BL/6	HFD	14/14	M (300 mg/kg) vs. non-treatment	12 w	Ceca	16S rRNA V3–V4 region	With vs. without	√	√	√		
Ji et al. (38), China	Male C57BL/6J	HFD	5/5	M (300 mg/kg) vs. non-treatment	3 w	Feces	16S rRNA V4 region	With vs. without	√	√	√		
Adeshirlarijaney et al. (41), USA	Male C57BL/6	HFD	10/NA	M (300 mg/kg IP) vs. vehicle	10 w	Feces	16S rRNA V4 region	With vs. without	√	√	√		
Liao et al. (60), China	Male C57BL/6	HFD	NA	A (400 mg/kg) vs. Si (4 g/kg) vs. Sa (300 mg/kg) vs. L (200 μg/kg) vs. normal saline	4 w	Feces	16S rDNA V3–V4 region	With vs. without (Si)		√	√	√	
								With vs. without (A, Sa, L)		√			
Madsen et al. (65), Denmark	Male C57BL/6	HFD	15/15	L (0.4 mg/kg) vs. vehicle	28 d	Feces	16S rDNA V3–V4 region and metagenomics	Post vs. pre	√	√	√		
Wang et al. (45), Korea	Male C57BL/6J	HFD	16/8	M (100 mg/kg) vs. non-treatment	10 w	Feces	16S rRNA gene RT-qPCR	With vs. without		√	√		
Lee et al. (48), Korea	Male C57BL/6N	HFD	6/6	M (250 mg/kg) vs. non-treatment	16 w	Ceca	16S rRNA V4 region	With vs. without	√	√	√		
Zhou et al. (53), China	Male C57BL/6J	HFD	NA	M (100 mg/kg) vs. non-treatment	4 w	Feces	16S rRNA gene RT-qPCR	With vs. without			√		
Do et al. (63), Korea	Male C57BL/6J	HFD	9/10	Vo (1 mg/kg) vs. non-treatment	12 w	Feces	16S rRNA V1–V3 region	With vs. without			√		√
Lee and Ko (55), Korea	Female C57BL/6	ND, HFD	NA	M (300 mg/kg) vs. non-treatment	10 w	Feces	16S rRNA V1–V3 region	With vs. without	√	√	√		
Shin et al. (56), Korea	C57BL/6	ND, HFD	12/12	M (300 mg/kg) vs. non-treatment	6 w	Feces	16S rRNA gene RT-qPCR	With vs. without		√	√		
Dong et al. (39), USA	KC	Gene knockout with HFCD	8/8	M (5 mg/ml in drinking water) vs. non-treatment	2 m	Duodena, ilea, ceca	16S rRNA V4 region	With vs. without	√	√	√		
Brandt et al. (40), Germany	Female C57BL/6J	FFCD	6–8/6–8	M (300 mg/kg) vs. non-treatment	4 d	Proximal small intestine	16S rRNA V1–V2 region	With vs. without	√	√	√		
Baxter et al. (61), USA	Male C57BL/6	HSD, PPD	25/5	A (25, 400 mg/kg) vs. non-treatment	2 w	Feces	16S rRNA V4 region	With vs. without		√	√	√	
								Post vs. pre		√	√	√	
Kishida et al. (64), Japan	Male C57BL/6J	HFHSD	10/10	Mi 0.04% in diet vs. non-treatment	12 w	Feces	16S rRNA V3–V4 region	With vs. without	√	√	√		
Olivares et al. (74), Belgium	Male C57BL/6J	WD	9/9	Vi (0.6 mg/mL in drinking water) vs. non-treatment	8 w	Ceca	16S rRNA V5–V6 region	With vs. without	√	√	√	√	
Zheng et al. (44), China	Male C57BL/6J	HFD/STZ	48/8	M (75, 200 mg/kg) vs. normal saline	5 w	Feces	16S rRNA V3–V4 region	With vs. without		√	√		
Wang et al. (70), China	Male ApoE^−/−^	HFD ± STZ	20/20/20	L (0.4 mg/kg) vs. Sa (10 mg/kg) vs. non-treatment	8 w	Feces	16S rRNA V1–V3 region	With vs. without (L, Sa)	√	√	√		
Xue et al. (36), China	Female C57BL/6J	DHEA+HFD	10/10	M (1.9 g/kg) vs. normal saline	21 d	Feces	16S rDNA V3–V4 region	With vs. without		√	√		
Moreira et al. (68), Brazil	Male C57BL/6J and female *ob/ob*	ND, HFD, gene knockout	24–48/24–48	L (400 μg/kg) vs. normal saline	15 d	Feces	16S rRNA V3–V4 region	With vs. without	√		√		
Ma et al. (46), China	C57BL/6	ND	10/9	M (300 mg/kg) vs. normal saline	30 d	Feces	16S rRNA	With vs. without	√	√	√		
Xu et al. (62), China	Male ICR	ND	5/5/5	A (4 mg/kg) vs. Vo (0.008 mg/kg) vs. non-treatment	2 w	Intestine	16S rRNA V4 region	With vs. without			√	√	
Zhang et al. (35), China	BKSLeprdb (*db/db*)	Gene knockout	5/5	M (113.75 mg/kg) vs. non-treatment	11 w	Feces	16S rRNA V3–V4 region	With vs. without	√	√	√	√	
Lee et al. (76), USA	C57BLKS/J-leprdb/leprdb (*db/db*)	Gene knockout	12/12	D (60 mg/kg in diet) vs. non-treatment	8 w	Feces	16S rRNA V4 region	With vs. without	√	√	√		
Li et al. (75), China	Male ICR MafA-deficient	Gene knockout	8/8	D (1.0 mg/kg) vs. normal saline	6 w	Intestine and feces	16S rRNA V3–V4 region	With vs. without	√	√	√	√	
Li et al. (78), China	Female KKAy	Gene knockout	6/6	P vs. distilled water	NA	Feces	16S rDNA DGGE	With vs. without	√	√	√		
Wang et al. (79), China	KKAy	Gene knockout	7/6	R (2 mg/kg) vs. distilled water	8 w	Intestine	Cultivation	With vs. without			√		
Smith et al. (59), USA	Offsprings of female CByB6 mF1/J and male C3D2F1/J	ND	71/72	A (1,000 ppm with diet) vs. non-treatment	17–25 m	Feces	16S rRNA V4 region	With vs. without	√	√	√	√	
Salomäki-Myftari et al. (52), Finland	Offsprings of homozygous OE-NPY	Gene knockout	NA	M (300 mg/kg) vs. vehicle (for dams)	18 d	Feces	16S rRNA V4–V5 region	With vs. without		√	√		
Mishima et al. (77), Japan	Male C57BL/6	Adenine induced renal failure	8/8	C (10 mg/kg) vs. vehicle	2 w	Ceca	16S rRNA V1–V2 region	With vs. without		√	√	√	
**Studies in rats (*****n*** **=** **17)**													
Bauer et al. (43), Canada	Male SD	HFD	6/6	M (200 mg/kg) vs. normal saline	1 d	Lumina	16S rRNA V3 region	With vs. without	√	√	√		
Zhang et al. (54), China	Male W	HFD	10/10	M (200 mg/kg) vs. vehicle	8 w	Feces	16S rRNA V3 region	With vs. without	√	√	√		
Pyra et al. (57), Canada	Male SD	HFHSD	20/10	M (300 mg/kg) vs. non-treatment	7 w	Ceca	DNA gene RT-qPCR	With vs. without			√		
Dennison et al. (72), Canada	Female SD	HFHSD	11–13/11–13	Si (10 mg/kg) vs. non-treatment	12 w	Feces	16S rRNA gene RT-qPCR	With vs. without			√		
Liu et al. (47), China	Male W	HFD/STZ	10/10	M (200 mg/kg) vs. non-treatment	4 w	Colon	16S rRNA V3–V4 region	With vs. without	√	√	√		
Xu M et al. (42), China	SD	HFHSD/STZ	10/10	M (1.8 g/kg) vs. non-M	4 w	Feces	16S rDNA gene qPCR	With vs. without			√		
Zhang et al. (69), China	Male SD	HFD/STZ	6/6	L (0.4 mg/kg) vs. normal saline	NA	Feces	16S rRNA V3–V4 region	With vs. without	√	√	√		
Zhang et al. (6), China	Male SD	HFD/STZ	12/6	Vi (0.01, 0.02 g/kg) vs. vehicle	12 w	Feces	16S rRNA V3–V4 region	With vs. without	√	√	√		
Yan et al. (73), China	Male SD	HFHC/STZ	10/10	Si (10 mg/kg) vs. non-treatment	12 w	Feces	16S rRNA V1–V3 region	With vs. without	√	√	√		
Yuan et al. (67), China	Male SD	STZ	6/6	L (0.6 mg/kg) vs. non-L	NA	Feces	16S rRNA V3 region	With vs. without			√		
Zhang et al. (5), China	Male ZDF	Gene knockout	8/8/8/8	A (32.27 mg/kg) vs. M (215.15 mg/kg) vs. Si (10.76 mg/kg) vs. normal saline	4 w	Feces	16S rRNA V3–V4 region	A vs. M A vs. Si	√	√	√		
								With vs. without (A)	√	√	√		
								With vs. without (M, Si)		√	√		
Shin et al. (49), Korea	Male OLETF	Gene knockout	7/7	M (100 mg/kg) vs. water	12 w	Feces	16S rRNA V1–V3 region	With vs. without	√	√	√		
Wang et al. (50), Korea	Male OLETF	Gene knockout	NA	M (100 mg/kg) vs. distilled water	12 w	Feces	16S rRNA V3 region	With vs. without		√	√	√	
Han et al. (51), Korea	Male OLETF	Gene knockout	7/7	M (100 mg/kg) vs. distilled water	12 w	Feces	16S rRNA V1–V2 region	With vs. without			√		√
Zhao et al. (58), China	Male GK	Gene knockout	6/6	A (50 mg/kg) vs. normal saline	8 w	Colon, feces	16S rRNA V3–V4 region	With vs. without	√	√	√		
Zhao et al. (66), China	Male W and GK	HFD, gene knockout	16/16	L (400 μg/kg) vs. normal saline	12 w	Feces	16S rRNA V3–V4 region	With vs. without	√	√	√		
Kaya et al. (71), Japan	Male OLETF	Gene knockout and PS	10/10	An (45 mg/kg) vs. vehicle	8 w	Feces	16S rRNA V4 region	With vs. without	√		√		

*A, acarbose; An, anagliptin; C, canagliflozin; D, dapagliflozin; G, glipizide; Mi, miglitol; M, metformin; L, liraglutide; P, pioglitazone; R, rosiglitazone; Si, sitagliptin; Sa, saxagliptin; Vo, voglibose; Vi, vildagliptin; HFD, high-fat diet; ND, normal-chow diet; HFHSD, high-fat high-sucrose diet; HFHCD, high-fat high-carbohydrate diet; DHEA, trans-dehydroandrosterone; HFCD, high-fat high-calories diet; FFCD, fat-, fructose-, and cholesterol-rich diet; HSD, high-starch diet; PPD, plant plolysaccharide diet; WD, Western diet; STZ, streptozocin intraperitoneal injection; DHEA, trans-dehydroandrosterone; PS, pocrine serum intraperitoneal injection; IP, Intraperitoneal injection; SD rats, Sprague-Dawley rats; ZDF rats, Male Zucker diabetic fatty rats, induced by leptin receptor gene knockout; KC mice, LSL-KrasG12D/+ and p48-Cre+/- mice, induced by LSL-KRASG12D and Cre alleles knockout; OLETF rats, Otsuka Long-Evans Tokushima Fatty rats, induced by spontaneous CCK_1_ receptor knockout; OE-NPY mice, homozygous transgenic OE-NPY mice, induced by transgenic mice overexpressing Neuropeptide Y under dopamine–β-hydroxylase promoter; W rats, Wistar rats; GK rats, Goto-Kakizaki rats, induced by polygenic Wistar substrain; ob/ob mice, mice model induced by Lep^°b^ gene knockout; ICR MafA-deficient mice, model induced by targeted disruption of the mafA gene in ICR mice; db/db mice, model induced by mutation in the leptin receptor gene in mice; KKAy mice, induced by transfer the yellow obese gene (A^y^) into KK mice; RT-qPCR, Real-time quantitative polymerase chain reaction; DGGE, denaturing gradient gel electrophoresis; m, months; w, weeks; d, days; NA, not available; vs., versus*.

### Microbiome Assessment Method

Fecal specimens of all human studies were analyzed for the composition of gut microbiota. Of 47 mouse studies, 34 studies (72.3%) used fecal samples (5, 6, 35, 36, 38, 41, 42, 44–46, 49–56, 59–61, 63–73, 76, 78). 16S rRNA gene sequencing was the most common method used in human and animal studies (Table 1).

### The Risk of Bias

Among human studies, three randomized studies were at the high risk of bias in performance, detection, and attrition, while four studies were unclear risks in most domains (Supplementary Figure 1). Among quasi-experimental studies, three studies were at low risk of bias in all domains, whereas two studies were at serious risk in confounding, selection of participants, and classification of interventions (Supplementary Figure 2). All 5 cross-sectional studies were at serious risk in several domains, e.g., confounding, selection of participants, classification of intervention (Supplementary Figure 3). Almost all mouse studies were unclear risk across domains, even if some were at low risk of bias in selective outcome reporting (Supplementary Figure 4).

### Outcomes of Assessment

#### Bacterial Taxa

Importantly, the synthesized results from animal studies reported all common six taxonomic categories (P, C, O, F, G, S) of bacterial taxa but there were only three common taxonomic categories (F, G, S) of gut microbiota taxa were reported in those from human studies based on the available data (Tables 4–**7**). Glipizide and liraglutide were assessed in a single human study. No differences were found in patients treated with glipizide (32), while Wang et al. (34) found the association between liraglutide treatment and the increased abundance of genus *Akkermansia* in T2D patients. The assessments of the effects of metformin and acarbose on the human gut microbiota composition represented the foci of 14 studies (19–31, 34) and three studies (7, 32, 33), respectively (Tables 4, 5).

**Table 4:** Effects of anti-hyperglycemic drugs on specific taxa in human gut microbiota, categorized by the target research populations^a^.

**Specific taxa**	**Phylum**	***N*^b^**	***N*/*N*^c^**	**Healthy**	**Obese**	**Pre-diabetic**	**Newly T2D**	**Prevalent T2D**
G_*Alistipes*	Bacteroidetes	2	151/151				↓ A (32), M (20)	
G_*Bacteroides*	Bacteroidetes	5	233/233		↔ M (19)	↓ A (7)	↓ A (32), M (20, 24)	
S_*Bacteroides dorei*	Bacteroidetes	2	73/73				↓ A (32), M (24)	
S_*Bacteroides finegoldii*	Bacteroidetes	2	73/73				↓ A (32), M (24)	
S_*Bacteroides intestinalis*	Bacteroidetes	2	73/73				↓ A (32), M (24)	
S_*Bacteroides stercoris*	Bacteroidetes	2	73/73				↓ A (32), M (24)	
S_*Bacteroides thetaiotaomicron*	Bacteroidetes	2	73/73				↓ A (32), M (24)	
S_*Bacteroides uniformis*	Bacteroidetes	2	73/73				↓ A (32), M (24)	
S_*Bacteroides vulgatus*	Bacteroidetes	2	73/73				↓ A (32), M (24)	
G_*Bifidobacterium*	Actinobacteria	2	73/73				↑ A (32), M (21)	
S_*Bifidobacterium adolescentis*	Actinobacteria	2	73/73				↑ A (32), M (21)	
G_*Clostridium*	Firmicutes	2	76/76	↓ M (22)			↓ A (32)	
S_*Clostridium leptum*	Firmicutes	2	71/84				↓ A (32)	↓ M (31)
F_*Lachnospiraceae*	Firmicutes	2	140/140			↓ A (7)	↑ M (20)	
G_*Lactobacillus*	Firmicutes	3	111/111		↔ M (19)	↑ A (7)	↑ A (32)	
S_*Lactobacillus gasseri*	Firmicutes	2	73/73				↑ A (32), M (21)	
G_*Megasphaera*	Firmicutes	2	54/54			↑ A (7)		↑ M (29)
S_*Pseudoflavonifractor capillosus*	Firmicutes	2	73/73				↓ A (32), M (21)	
S_*Ruminococcus* sp. *5_1_39BFAA*	Firmicutes	2	73/73				↓ A (32), ↑ M (21)	

a *The target research populations include obese, pre-diabetic, newly Type 2 diabetes (T2D), prevalent T2D*;

b *Number of studies*;

c *Number of participants (treatment/comparison); F, family; G, genus; S, species; M, metformin; A, acarbose; ↑, significant increase; ↓, significant decrease; ↔, no significant difference*.

**Table 5:** Consistent and inconsistent effects of each anti-hyperglycemic drug on specific taxa in human gut microbiota, categorized by the target research populations^a^.

**Drug/specific taxa**	**Phylum**	***N*^b^**	***N*/*N*^c^**	**Healthy**	**Obese**	**Pre-diabetic**	**Newly T2D**	**Prevalent T2D**
**METFORMIN**								
**Consistent results**								
G_*Fusobacterium*	Fusobacteria	2	151/126				↑ (20, 27)	
S_*Akkermansia muciniphila*	Verrucomicrobia	2	36/36				↑ (21)	↑ (29)
G_*Escherichia*	Proteobacteria	6	243/202	↑ (22, 25)	↑ (19)		↑ (20, 21)	↑ (30)
G_*Shigella*	Proteobacteria	4	163/163	↑ (22, 25)	↑ (19)		↑ (20)	
S_*Citrobacter koseri*	Proteobacteria	2	42/55				↑ (21)	↑ (31)
S_*Citrobacter rodentium*	Proteobacteria	2	42/55				↑ (21)	↑ (31)
S_*Enterobacter cloacae*	Proteobacteria	2	42/55				↑ (21)	↑ (31)
S_*Escherichia coli*	Proteobacteria	2	42/55				↑ (21)	↑ (31)
S_*Klebsiella pneumonia*	Proteobacteria	2	42/55				↑ (21)	↑ (31)
S_*Erwinia amylovora*	Proteobacteria	2	42/55				↑ (21)	↑ (31)
S_*Pectobacterium wasabiae*	Proteobacteria	2	42/55				↑ (21)	↑ (31)
S_*Dickeya dadantii*	Proteobacteria	2	42/55				↑ (21)	↑ (31)
S_*Clostridium bartlettii*	Firmicutes	2	42/55				↓ (21)	↓ (31)
S_*Clostridium botulinum*	Firmicutes	2	42/55				↓ (21)	↓ (31)
**Inconsitent results**								
G_*Bacteroides*	Bacteroidetes	3	142/142		↔ (19)		↓ (20, 24)	
G_*Prevotella*	Bacteroidetes	2	34/34		↔ (19)			↑ (29)
G_*Blautia*	Firmicutes	3	138/138	↔ (25)	↔ (19)		↑ (20)	
G_*Intestinibacter*	Firmicutes	4	125/84	↓ (22)	↔ (19)		↓ (21)	↓ (30)
F_*Enterobacteriaceae*	Proteobacteria	2	69/44	↑ (25)				↓ (27)
**Acarbose**								
**Consistent results**								
G_*Bacteroides*	Bacteroidetes	2	91/91			↓ (7)	↓ (32)	
G_*Lactobacillu*s	Firmicutes	2	91/91			↑ (7)	↑ (32)	
S_*Bidifobacterium longum*	Actinobacteria	2	110/110				↑ (32)	↑ (33)

a *The target research populations include obese, pre-diabetic, newly Type 2 diabetes (T2D), prevalent T2D*;

b *Number of studies*;

c *Number of participants (treatment/comparison); F, family; G, genus; S, species; ↑, significant increase; ↓, significant decrease; ↔, no significant difference*.

Of the phylum *Bacteroidetes*, the genus *Bacteroides* decreased in two studies treated with metformin among newly diagnosed T2D patients (20, 24), and in two studies treated with acarbose among pre-diabetic and newly diagnosed T2D patients (7, 32). Additionally, one study treated with metformin (24) and one study treated with acarbose (32) in newly diagnosed T2D patients reported similar results of decreases in seven species (e.g., *Bacteroides dorei, Bacteroides finegoldii*). For the phylum *Firmicutes*, the genus *Lactobacillus* increased in two studies in pre-diabetic (7) and newly diagnosed T2D patients (32) receiving acarbose, and the species *L. gasseri* increased in one study treated with metformin (21), and with acarbose (32), respectively, among newly diagnosed T2D patients. Meanwhile, the genus *Clostridium* decreased in one study among healthy participants receiving metformin (22) which was also reported in newly diagnosed T2D patients treated with acarbose (32) (Table 4). Two species, i.e., *C. bartlettii* and *C. botulinum*, consistently decreased among T2D patients receiving metformin in two separate studies (21, 31). Three out of four studies showed a decrease in the genus *Intestinibacter* (21, 22, 30) among healthy participants and T2D patients treated with metformin (Table 5). With respect to the phylum *Actinobacteria*, the genus *Bifidobacterium* with the species *B. adolescentis* increased in one study with metformin (21) and another study with acarbose (32) among newly diagnosed T2D patients (Table 4), and *B. longum* consistently increased among T2D patients treated with acarbose in two studies (32, 33) (Table 5).

Concerning other phyla, two studies evaluated the genus *Fusobacterium* (phylum *Fusobacteria*) (20, 27) and the species *Akkermansia muciniphila* (phylum *Verrucomicrobia*) (21, 29) among T2D patients treated with metformin. Both showed increases in the abundance of these two taxa. In the phylum *Proteobacteria*, conflicting results were reported in two studies with respect to the family *Enterobacteriaceae* in healthy participants vs. T2D patients treated with metformin (25, 27). Overall, metformin might increase different taxa from the family *Enterobacteriaceae* and other families in the order of *Enterobacteriales*. Of six studies that evaluated the genus *Escherichia* (19–22, 25, 30) and four studies evaluating the genus *Shigella* (19, 20, 22, 25), the treatment of healthy participants, obese individuals, and T2D patients with metformin all led to increased abundance of these two genera. Another two studies on metformin in newly diagnosed and prevalent T2D patients (21, 31) reported consistently increases in eight species of the order *Enterobacteriales*, including *Citrobacter koseri, Escherichia coli, Klebsiella pneumonia* (family *Enterobacteriaceae*)*, Erwinia amylovora* (family *Erviniaceae*)*, Pectobacterium wasabiae*, and *Dickeya dadantii* (family *Pectobacteriaceae*) (Table 5).

The effects of 13 anti-hyperglycemic drugs on the compositions of gut microbiota were conducted in different rodent models. The results of pioglitazone was inconclusive (78), while the other 12 drugs were presented in Tables 6, 7. The *Firmicutes/Bacteroidetes* ratios were decreased in two studies treated with metformin in high fat diet (HDF)-fed mice (37, 48). This decrease was also noted in another two studies, in which liraglutide was given to in rat models induced by diets and gene knockout (66, 69).

**Table 6:** Effects of anti-hyperglycemic drugs on gut microbiota in mouse models, categorized by the treated, individual anti-hyperglycemic drug.

**Specific taxon**	***N*^a^**	**M**	**A**	**Mi**	**Vo**	**L**	**An**	**Sa**	**Si**	**Vi**	**C**	**D**	**R**
***Firmicutes/bacteroidetes***	9	↓ (2)	↔		↓	↓ (2)	↓			↓		↓	
**Phylum** ***Bacteroidetes***													
P_*Bacteriodetes*	15	↔(3), ↓, ↑ (5)	↔(2)			↓, ↑ (2)		↓	↔, ↑ (2)	↑		↑	
C_*Bacteroidia*	3	↑				↓, ↑							
F_*Bacteroidaceae*	5	↑	↑			↓			↑	↑			
G_*Bacteroides*	18	↔(2), ↓, ↑ (5)	↑		↑	↔, ↑, ↓	↑	↓	↑ (2)	↔, ↑		↑	
S_*Bacteroides acidifaciens*	2					↑				↑		↑	
G_*Butyricimonas*	3	↑ (2)				↑							
F_*Porphyromonadaceae*	4	↑ (2)				↓, ↑							
G_*Odoribacter*	4	↔, ↓ (2)	↔										
G_*Parabacteroides*	8	↓, ↑ (4)	↔			↓			↑				
F_*Prevotellaceae*	5	↑ (2)							↔,↑	↓			
G_*Prevotella*	8	↓, ↑ (3)				↓	↓	↓		↔			
G_*Prevotella_9*	3					↓, ↑				↑			
F_*Rikenellaceae*	3	↑ (2)		↑									
G_*Alistipes*	3	↑	↑		↑							↑	
F_*S24-7*	3		↓, ↑						↑				
**Phylum** ***Firmicutes***													
P_*Firmicutes*	15	↔(4), ↓ (3), ↑ (2)	↔, ↑			↔, ↓, ↑		↑	↓, ↑	↓		↑	
G_*Turicibacter*	5	↔, ↓, ↑	↔			↑		↑					
O_*Lactobacillales*	2	↓				↑							
G_*Enterococcus*	7	↔, ↓ (3), ↑										↓, ↑	
F_*Lactobacillaceae*	5	↓, ↑	↓ (2)			↑							
G_*Lactobacillus*	13	↓ (2), ↑ (6)	↔, ↓, ↑			↓, ↑		↑	↓, ↑				
S_*Lactobacillus intesinalis*	1	↑	↑						↑				
S_*Lactobacillus johnsonii*	2	↑							↑	↔			
F_*Streptococcaceae*	5	↓ (2)				↓			↓	↑			
G_*Streptococcus*	4	↓	↔							↑			↓
C_*Clostridia*	2	↓				↓							
O_*Clostridiales*	2	↓				↓							
F_*Clostridiales_vadinBB60_g*	2					↑			↓				
F_*Christensenellaceae*	3	↑				↓				↓			
G_*Christensenellaceae R_7_g*	3		↔			↔, ↓				↓			
G_*Candidatus Arthromitus*	3	↔	↔			↓							
G_*Clostridium*	3	↓				↑			↔				
G_*Ruminiclostridium*	2		↔						↓				
G_*Ruminiclostridium 6*	2		↓		↓	↓				↓			
G_*Ruminiclostridium 9*	3		↔			↔			↓				
F_*Ruminococcaceae*	6	↑	↓			↔, ↑			↓	↓			
G_*Ruminococcaceae_UCG_005*	1		↓		↓								
G_*Ruminococcus*	5	↓ (2), ↑ (2)							↑				
G_*Ruminococcus 2*	2		↓, ↑		↑								
F_*Lachnospiraceae*	5	↓	↔			↔, ↓			↓				
G_*Blautia*	8	↓, ↑	↑		↑	↑ (2)			↓ (2)			↑	
G_*Lachnoclostridium*	3	↓	↔			↑							
G_*Lachnospiraceae_nk4a136_g*	2					↔						↑	
G_*Marvinbryantia*	2		↔			↓							
G_*Roseburia*	7	↔, ↓	↔			↔, ↓			↔, ↓				
G_*Peptococcus*	2	↓	↔										
G_*Romboustia*	2	↓				↓							
G_*Anaerotruncus*	6	↔, ↓	↔			↓			↓	↓			
G_*Flavonifractor*	2	↓				↑							
G_*Oscillospira*	6	↓, ↑				↑				↓	↓	↓	
F_*Dehalobacteriaceae*	3	↓, ↑		↑									
F_*Erysipelotrichaceae*	5	↑	↔, ↑	↓		↑							
O_*Erysipelotrichales*	2	↑				↑							
**Phylum** ***Actinobacteria***													
P_*Actinobacteria*	5	↔	↑			↔, ↓					↓		
F_*Bifidobacteriaceae*	2	↓	↑										
G_*Bifidobacterium*	8	↔(2), ↑, ↓	↑			↑			↑		↓		
S_*Bifidobacterium* spp.	2	↓								↔			
G_*Corynebacterium 1*	2		↔						↑				
G_*Enterorhabdus*	2		↔			↓							
**Phylum** ***Cyanobacteria***													
P_*Cyanobacteria*	2	↓	↓			↑			↓				
**Phylum** ***Elusimicrobia***													
P_*Elusimicrobia*	2					↔				↓			
G_*Allobaculum*	10	↔, ↓ (2), ↑ (2)	↑			↔, ↑		↑ (2)					
**Phylum** ***Fusobacteria***													
P_*Fusobacteria*	3	↓, ↑				↔							
**Phylum** ***Proteobacteria***													
P_*Proteobacteria*	8	↓ (3), ↑	↓			↔, ↓ (2)			↓↑			↑	
C_*Alphaproteobacteria*	2	↓								↑			
G_*Desulfovibrio*	5	↔, ↓	↓		↓	↑			↓				
F_*Desulfovibrionaceae*	4	↓	↓	↑					↓				
F_*Enterobacteriacae*	2	↑										↓	
G_*Escherichia*	3	↔, ↑											↓
G_*Helicobacter*	2	↓				↓							
**Phylum** ***Tenericutes***													
P_*Tenericutes*	6	↔, ↑	↔			↑ (2)			↔, ↑	↓			
C_*Mollicutes*	2	↑				↓							
**Phylum** ***Verrucomicrobia***													
P_*Verrucomicrobia*	8	↔, ↓ (2), ↑ (5)	↓			↑							
F_*Verrucomicrobiaceae*	5	↑ (4)	↓ (2)										
G_*Akkermansia*	9	↑ (8)				↑							
S_*Akkermansia muciniphila*	6	↔, ↑ (3)				↑ (2)							

a *Number of studies; M, metformin; A, acarbose; Mi, miglitol; Vo, voglibose; L, liraglutide; An, anagliptin; Sa, saxagliptin; Si, sitagliptin; Vi, vildagliptin; C, canagliflozin; D, dapagliflozin; R, rosiglitazone; P, Phylum; C, Class; O, Order; F, Family; G, Genus; S, Species; ↑, significant increase; ↓, significant decrease; ↔, no significant difference; (n), Number of studies (≥2) reported the same results*.

**Table 7:** Consistent and inconsistent effects of each anti-hyperglycemic drug on specific taxa in mouse gut microbiota, categorized by mice or rat models with three distinct animal models.

**Specific taxa**	**Phylum**	***N*^a^**	**Trend^b^**	**Mice models**			**Rat models**		
				**Normal**	**Dietary or STZ**	**Gene knockout**	**Normal**	**Dietary or STZ**	**Gene knockout**
**METFORMIN**									
**Consistent results**									
*Firmicutes/Bacteroidetes*		2	↓		↓ (2)				
F_*Porphyromonadaceae*	Bacteroidetes	2	↑	↑	↑				
F_*Prevotellaceae*	Bacteroidetes	2	↑	↑				↑	
F_*Rikenellaceae*	Bacteroidetes	2	↑	↑ (2)					
G_*Butyricimonas*	Bacteroidetes	2	↑		↑	↑			
F_*Enterococcaceae*	Firmicutes	2	↓		↓			↓	
F_*Streptococcaceae*	Firmicutes	2	↓		↓			↓	
F_*Verrucomicrobiaceae*	Verrucomicrobia	4	↑	↑ (2)	↑ (3)				
G_*Akkermansia*	Verrucomicrobia	8	↑		↑ (6)	↑		↑	
S_*Akkermansia* spp.	Verrucomicrobia	2	↑	↑					↑
F_*Alcaligenaceae*	Proteobacteria	2	↑		↑			↑	
**Inconsistent results**									
P_*Bacteroidetes*	Bacteroidetes	8	↑	↔	↓, ↑ (4)			↔, ↑	↔
G_*Bacteroides*	Bacteroidetes	7	↑	↑	↔, ↓, ↑ (2)	↔		↑ (2)	
G_*Odoribacter*	Bacteroidetes	3	↓		↓	↔			↓
G_*Parabacteroides*	Bacteroidetes	5	↑	↓	↓, ↑ (4)				
G_*Prevotella*	Bacteroidetes	4	↑		↑	↓		↑ (2)	
P_*Firmicutes*	Firmicutes	8	°	↔	↔, ↑, ↓ (3)			↔(2)	↑
G_*Allobaculum*	Firmicutes	5	°	↓	↑, ↓	↔		↑	
G_*Anaerotruncus*	Firmicutes	2	°		↓	↔			
G_*Blautia*	Firmicutes	2	°		↓			↑	
G_*Christensenella*	Firmicutes	2	°		↑	↔			
G_*Coprococcus*	Firmicutes	3	↓		↓ (2)	↑			
F_*Dehalobacteriaceae*	Firmicutes	2	°		↓				↑
G_*Dehalobacterium*	Firmicutes	2	°		↓				↑
G_*Enterococcus*	Firmicutes	5	↓		↓ (2)			↔, ↑, ↓	
F_*Lactobacillaceae*	Firmicutes	2	°					↑, ↓	
G_*Lactobacillus*	Firmicutes	8	↑		↑, ↓	↑		↓, ↑ (3)	↑
G_*Lactococcus*	Firmicutes	2	°		↑, ↓				
G_*Oscillospira*	Firmicutes	2	°		↑, ↓				
G_*Roseburia*	Firmicutes	2	°			↔		↓	
G_*Ruminococcus*	Firmicutes	4	°	↑	↓ (2)	↑			
G_*Turicibacter*	Firmicutes	3	°	↑		↔, ↓			
G_*Bifidobacterium*	Actinobacteria	4	°		↔	↔		↑, ↓	
P_*Fusobacteria*	Fusobacteria	2	°		↑				↓
C_*Fusobacteriia*	Fusobacteria	2	°		↑				↓
O_*Fusobacteriales*	Fusobacteria	2	°		↑				↓
P_*Proteobacteria*	Proteobacteria	4	↓		↓			↑, ↓	↓
G_*Desulfovibrio*	Proteobacteria	2	°		↓	↔			
G_*Escherichia*	Proteobacteria	2	°		↑			↔	
S_*Escherichia coli*	Proteobacteria	2	°		↓			↔	
G_*Klebsiella*	Proteobacteria	2	°					↔, ↑	
G_*Parasutterella*	Proteobacteria	3	↑	↑	↔			↑	
G_*Proteus*	Proteobacteria	2	°		↑	↓			
G_*Sutterella*	Proteobacteria	3	↑		↑	↔		↑	
G_*Trabulsiella*	Proteobacteria	2	°		↓	↔			
P_*Tenericutes*	Tenericutes	2	°		↑				↔
P_*Verrucomicrobia*	Verrucomicrobia	7	↑	↔	↑ (4)			↑, ↓	↓
S_*Akkermansia muciniphila*	Verrucomicrobia	4	↑		↔, ↑ (3)				
G_*AF12*	NA	2	°		↓	↔			
**ACARBOSE**									
**Consistent result**									
F_*Lactobacillaceae*	Firmicutes	2	↓	↓	↓				
**Inconsistent results**									
F_*S24-7*	Bacteroidetes	2	°	↑	↓				
P_*Firmicutes*	Firmicutes	2	°			↔			↑
F_*Erysipelotrichaceae*	Firmicutes	2	°	↔	↑				
G_*Lachnospiraceae UCG-001*	Firmicutes	2	°	↔					↑
G_*Lactobacillus*	Firmicutes	3	°	↔		↑			↓
G_*Ruminococcus 2*	Firmicutes	2	°	↓					↑
**DAPAGLIFLOZIN**									
**Inconsistent result**									
G_*Enterococcus*	Firmicutes	2	°			↑, ↓			
**LIRAGLUTIDE**									
**Consistent results**									
*Firmicutes/Bacteroidetes*		2	↓				↓	↓	↓
G_*Blautia*	Firmicutes	2	↑		↓				↓
S_*Akkermansia muciniphila*	Verrucomicrobia	2	↑		↑ (2)				
*Inconsistent results*									
P_*Actinobacteria*	Actinobacteria	2	°		↓			↔	↔
P_*Bacteroidetes*	Bacteroidetes	3	↑		↓			↑ (2)	↑
G_*Bacteroides*	Bacteroidetes	3	°		↓			↑	↔
C_*Bacteroidia*	Bacteroidetes	2	°		↓			↑	↑
O_*Bacteroidales*	Bacteroidetes	2	°		↓			↑	
F_*Porphyromonadaceae*	Bacteroidetes	2	°		↓			↑	
G_*Prevotella_9*	Bacteroidetes	2	°					↓, ↑	↑
P_*Firmicutes*	Firmicutes	3	°		↑			↔, ↓	↔
G_*Allobaculum*	Firmicutes	2	°		↑				↔
G_*Christensenellaceae _R_7_group*	Firmicutes	2	°					↔, ↓	↔
F_*Lachnospiraceae*	Firmicutes	2	°		↓			↔	↔
F_*Ruminococcaceae*	Firmicutes	2	°					↔, ↑	↔
G_*Lactobacillus*	Firmicutes	2	°		↑			↓	↔
G_*Roseburia*	Firmicutes	2	°		↓				↔
P_*Proteobacteria*	Proteobacteria	3	↓	↓	↓ (2)	↓		↔	↔
P_*Tenericutes*	Tenericutes	2	°					↔, ↓	↔
P_*Verrucomiacrobia*	Verrucomiacrobia	2	°	↑	↑			↔	↔
**SITAGLIPTIN**									
**Consistent results**									
G_*Bacteroides*	Bacteroidetes	2	↑		↑			↑	
G_*Blautia*	Firmicutes	2	↓		↓			↓	
**Inconsistent results**									
P_*Bacteroidetes*	Bacteroidetes	2	°		↑				↔
F_*Prevotellaceae*	Bacteroidetes	2	°		↑			↔	
P_*Firmicutes*	Firmicutes	2	°					↓	↑
G_*Lactobacillus*	Firmicutes	2	°					↓	↑
G_*Roseburia*	Firmicutes	2	°		↓			↔	
P_*Proteobacteria*	Proteobacteria	2	°					↑	↓
P_*Tenericutes*	Tenericutes	2	°					↑	↔
**VILDAGLIPTIN**									
**Consistent result**									
G_*Oscillibacter*	Firmicutes	2	↓		↓				
**Inconsistent result**									
G_*Bacteroides*	Bacteroidetes	2	°		↔			↑	

*P, Phylum; C, Class; O, Order; F, Family; G, Genus*;

a *Number of studies*;

b *Trend of alteration (reported in > 50% of studies): ↑, a trend of increase; ↓, a trend of decrease; °, inconclusive results; Alteration of specific taxa: ↑, significant increase; ↓, significant decrease; ↔, no significant difference; (n), number of papers (≥2) reported the same results*.

Responses of the phylum *Bacteroidetes* to different anti-hyperglycemic agents were investigated in 15 rodent studies. No difference was noted in studies treated with acarbose (5, 58). Five out of the seven studies on the genus *Bacteroides* after using metformin (37, 42, 48, 54, 56) revealed increased abundance of this genus in mice and rats.

Among 15 rodent studies (5, 6, 36, 37, 41, 47, 48, 54, 56, 58, 66, 69, 70, 73, 75) on the phylum *Firmicutes*, the results were inconclusive among those treated with metformin (5, 36, 37, 41, 47, 48, 54, 56), acarbose (5, 58), liraglutide (66, 69, 70), and sitagliptin (5, 73). The genus *Lactobacillus* was the focus of 13 studies (5, 35, 41–43, 47, 53, 54, 58, 62, 66, 70, 73). Six out of the eight studies treated with metformin (5, 35, 43, 53, 54, 62) saw an increase in this genus in mice and rats, while the results in studies treated with acarbose (5, 58, 62), liraglutide (66, 70), and sitagliptin (5, 73) were inconclusive.

With respect to other phyla, there was a trend of decrease in the phylum *Proteobacteria* in mice treated with metformin and liraglutide, while *Verrucomicrobia* and *Tenericutes* increased after treated with metformin and liraglutide, respectively. However, results for the phyla *Actinobacteria, Cyanobacteria, Elusimicrobia*, and *Fusobacteria* were conflicting. The genus *Akkermanisa* (phylum *Verrucomicrobia*) increased in eight studies treated with metformin using dietary or genetic models (35, 37, 38, 41, 44, 48, 54, 56). Three of the four studies with metformin (41, 53, 55) reported an increase in the species *A. muciniphila*, and another two studies reported a similar increase in this species after treating with liraglutide (65, 68).

#### Diversity

Ten human studies treated with metformin (19, 20, 22–25, 27–30) and two studies treated with acarbose (7, 32) have provided the results of α-diversity. The results from those metformin studies, however, were conflicting, while both acarbose studies reported a decrease in the α-diversity among pre-diabetic and T2D patients. β-diversity was assessed in ten studies treated with metformin (19–22, 24, 25, 27–29, 34), of which six studies (20–22, 24, 27, 34) revealed a significant difference after the treatment in healthy participants and T2D patients. This difference was also noted in pre-diabetic patients treated with acarbose (32), and T2D patients treated with liraglutide (34) (Table 8).

**Table 8:** Effects of anti-hyperglycemic drug on diversity of human gut microbiota.

**Drugs**	**Object**	**α-diversity**		**β-diversity^c^**	**References**
		**Richness^a^**	**Evenness^b^**		
Metformin	Healthy	–	↓	ns	(25)
		ns	ns	≠	(22)
	Obese	ns	ns	ns	(19)
	Newly T2D	ns	↑	≠	(20)
		–	↓	≠	(24)
	Prevalent T2D	–	–	≠	(21)
		ns	ns	–	(23)
		–	↓	≠	(27)
		–	ns	ns	(28)
		ns	–	ns	(29)
		ns	–	–	(30)
		–	–	≠	(34)
Acarbose	Pre-diabetic	ns	↓	≠	(7)
	Newly T2D	↓	↓	–	(32)
Liraglutide	Prevalent T2D	–	–	≠	(34)
Glipizide	Newly T2D	ns	ns	–	(32)

a *Richness was assessed by Chao1, ACE, and Rarefaction indices, gene count, number of OTUs, or number of species*;

b *Evenness was assessed by Shannon, Simpson indices*;

c *β-diversity was assessed by UniFrac (weighted, unweighted), Bray-Curtis, Jensen-Shannon, or Jaccard distances using Principal Component Analysis (PCA) and Principal Coordinates Analysis (PCoA); ↑, significant increase; ↓, significant decrease; ≠, significant difference; ns, no significant difference; –, no information*.

Similar results with metformin and acarbose were reported in mouse studies. The effects of metformin on α-diversity were conflicting across different models, while the α-diversity decreased consistently in three studies treated with acarbose (5, 58, 59). Moreover, the results were inconsistent among those studies treated with liraglutide, sitagliptin, vildagliptin, and saxagliptin. In terms of β-diversity, there was higher cumulative evidence of significant difference after using metformin (5, 36–39, 41, 43–50, 54–56), and similar results were consistently reported among those studies treated with acarbose (5, 58–61), liraglutide (60, 65, 66, 69, 70), sitagliptin (5, 60, 73), and vildagliptin (6, 74) across different mouse models. Evidence for the effects of other drugs was limited (Table 9).

**Table 9:** Effects of anti-hyperglycemia drugs on diversity in mouse gut microbiota.

**Drugs**	**Objects**	**Models**	**α-diversity**		**β-diversity^c^**	**References**
			**Richness^a^**	**Evenness^b^**		
Metformin	Mice	ND	–	ns	≠	(46)
			ns	–	ns	(55)
			–	ns	ns	(40)
			–	–	ns	(56)
		HFD	–	↓	≠	(37)
			–	ns	≠	(38)
			ns	–	≠	(41)
			–	–	≠	(45)
			ns	ns	≠	(48)
			↓	–	≠	(55)
					≠	(56)
		HFCD	ns	ns	≠	(39)
		FFCD	–	ns	ns	(40)
		HFD/STZ	–	–	≠	(44)
		DHEA/HFD	–	–	≠	(36)
		*db/db*	ns	↑	ns	(35)
		OE-NPY	–	–	ns	(52)
	Rats	HFD	ns	ns	≠	(43)
		HFD/STZ	↓	↓	≠	(54)
			↑	ns	≠	(47)
		OLETE	↑	–	≠	(49)
			–	–	≠	(50)
		ZDF	–	–	≠	(5)
Acarbose	Mice	ND	↓	↓	≠	(59)
		HSD	–	–	≠	(61)
		HFD	–	–	≠	(60)
	Rats	GK	–	↓	≠	(58)
		ZDF	↓	↓	≠	(5)
Miglitol	Mice	HFHSD	–	ns	≠	(64)
Liraglutide	Mice	ND	↓	–	–	(68)
		HFD	ns	ns	≠	(65)
			↑	–	–	(68)
			ns	↓	≠	(70)
			–	–	≠	(60)
		HFD/STZ	ns	ns	≠	(70)
		*ob/ob*	↑	–	–	(68)
	Rats	HFD/STZ	↓	↓	≠	(69)
		GK	ns	ns	≠	(66)
		W	↓	↓	≠	(66)
Sitagliptin	Mice	HFD	–	–	≠	(60)
	Rats	HFHC/STZ	↑	↑	≠	(73)
		ZDF	–	–	≠	(5)
Vildagliptin	Mice	WD	ns	ns	≠	(74)
		HFD/STZ	↓	↓	≠	(6)
Saxagliptin	Mice	HFD	–	–	≠	(60)
			ns	↓	ns	(70)
		HFD/STZ	ns	ns	ns	(70)
Anagliptin	Rats	OLETF & PS	ns	ns	–	(71)
Dapagliflozin	Mice	MafA-deficient	↓	↓	ns	(75)
	Mice	*db/db*	ns	ns	ns	(76)
Canagliflozin	Mice	Adenine	–	–	≠	(77)
Pioglitazone	Mice	KKAy	–	↓	≠	(78)

a *Richness was assessed by Chao1, ACE, and Rarefaction indices, gene count, number of OTUs, or number of species*;

b *Evenness was assessed by Shannon, Simpson indices*;

c *β-diversity was assessed by UniFrac (weighted, unweighted), Bray-Curtis, Jensen-Shannon, or Jaccard distances using Principle Component Analysis (PCA) and Principle Coordinates Analysis (PCoA); ↑, significant increase; ↓, significant decrease; ≠, significant difference; ns, no significant difference; –, no information*.

#### Short-Chain Fatty Acids (SCFAs)

In human studies, changes in the levels of three main SCFAs (acetate, propionate, and butyrate) in feces and sera were reported in three studies treated with metformin (19, 21, 23) (Supplementary Table 3). Wu et al. (21) found that the levels of fecal butyrate and propionate increased in T2D male patients. However, no difference in fecal levels of these two SCFAs was noted among obese women in Ejtahed's study (19). In contrast, fecal acetate levels decreased in obese women (19) did not change among T2D patients in Wu's study (21). Huang et al. (23) reported that the serum levels of all three SCFAs remained unchanged after treating with metformin in T2D patients.

In mouse studies, fecal levels of SCFAs after metformin interventions were assessed in *db/db* mice (35) and OLETF rats (50) (Supplementary Table 3). It was found that levels of acetate and butyrate increased, but propionate levels remained unchanged. The effects of acarbose on these SCFAs levels were also assessed in dietary models (59, 61, 62). These studies showed consistent results of increased levels of butyrate in feces and ceca, but the levels of acetate and propionate varied in a diet-dependent manner.

#### Bile Acids

In human studies, three clinical trials treated with metformin (21, 24, 26) and one randomized trial with acarbose and glipizide (32) were carried out in T2D patients to assess the respective effects on the fecal and serum levels of bile acids (Supplementary Table 4). Regarding metformin, one study (24) showed increases in the fecal level of conjugated secondary bile acids, while no difference was reported in the other two studies (21, 26). Two of these three studies (21, 24) reported increases in the blood level of secondary bile acids, while the other one (26) revealed an inverse trend. Concerning changes in total and primary bile acids, their levels in feces were unchanged among these three trials, but results in blood levels were conflicting (21, 26). The random trial assessing the effects of acarbose and glipizide on bile acid levels in newly diagnosed T2D patients (32) showed that acarbose might increase the plasma and fecal levels of primary bile acids, accompanied by decreases in secondary bile acids. In contrast, no significant changes in bile acid levels were found in patients treated with glipizide.

As for rodent studies, one study in rats (51) revealed that the fecal level of total bile acids increased while the levels in liver tissues were decreased after metformin intervention. One study in mice (63) found that voglibose treatment was associated with increases in serum levels of primary bile acids, accompanied by decreases in serum levels of secondary bile acids (Supplementary Table 4).

#### Associations With Host Metabolic Parameters

Among pre-diabetic and T2D patients treated with metformin (20, 21) or acarbose (7, 32, 33), alterations in certain specific taxa in human gut microbiota were associated with improvement in HbA1C and fasting blood glucose values, body weights, and lipid profiles (Table 10). For instance, increments in the genera *Escherichia, Shigella, Subdoligranulum*, and *Dialister*, and the species *Bifidobacterium adolescentis, Bifidobacterium longum*, and *Lactobacillus gasseri* were inversely associated with HbA1C after treating with metformin or acarbose (7, 20, 21, 32). In addition, there were inverse associations between increases in the genus *Blautia* and fasting blood glucose after treating with metformin (20).

**Table 10:** Association between specific taxa and human metabolic parameters.

**Parameters**	**Association**	**Specific taxa**	**Alteration**	**Drugs**	**Participants**	**References**
HbA1C	Negative	G_*Escherichia* G_*Shigella*	↑	M	Newly T2D	(20)
		S_ *Bifidobacterium adolescentis*	↑	M	Newly T2D	(21)
		S_*Lactobacillus gasseri* S*_Bifidobacterium longum*	↑	A	Newly T2D	(32)
		G_*Subdoligranulum* G*_Dialister*	↑	A	Pre-diabetic	(7)
Fasting blood glucose	Negative	G_*Blautia*	↑	M	Newly T2D	(20)
Body weight	Positive	S_*Bacteroides plebeius* S*_Bacteroides dorei* S*_Bacteroides vulgatus* S*_Clostridium bolteae*	↓	A	Newly T2D	(32)
	Negative	S_*Lactobacillus gasseri* S*_Bifidobacterium longum*	↑	A	Newly T2D	(32)
HDL cholesterol	Positive	S_*Bidifobacterium longum*	↑	A	Prevalent T2D	(33)
LDL cholesterol	Negative	G_*Blautia*	↑	M	Newly T2D	(20)

*G, genus; S, species; M, metformin; A, acarbose; ↑, significant increase; ↓, significant decrease*.

Mouse studies treated with metformin (44, 50, 55, 57), liraglutide (65, 66, 70), and saxagliptin (70) also explored the relationship between changes in the compositions of gut microbiota and improvement in various metabolic parameters (Table 11). It was found that some related specific taxa after treating with metformin in mice (i.e., *Bacteroides* spp., *Blautia*) were different from that in humans.

**Table 11:** Association between specific taxa and mouse metabolic parameters.

**Parameters**	**Association**	**Specific taxa**	**Alteration**	**Drugs**	**Models**	**References**
Fasting blood glucose	Positive	S_*Bacteroides* spp.	↓	M	HFD/STZ mice	(44)
	Negative	S_*Akkermansia muciniphila*	↑	M	HFD mice	(55)
		S_*Bifidobacterium* spp.	↓	M	HFHSD rats	(57)
Body weight	Positive	G_*Candidatus Arthromitus* G_*Roseburia* G_*Marvinbryantia*	↓	L	HFD/STZ mice	(70)
		S_*Clostridia* sp., S_*Clostridiales* spp. S_*Oscillospiraceae* sp. S_*Erysipelatoclostridium* sp. S_*Anaerotruncus* sp. *G3(2012)* S_*Firmicutes* sp. S_*Bacteroidales* sp.	↓	L	HFD mice	(65)
	Negative	S_*Clostridiales* spp. S_*Oscillospiraceae* sp. S_*Burkholderiales bacterium YL45* S_*Akkermansia muciniphila*	↑	L	HFD mice	(65)
		G_*Lactobacillus* G_*Turicibacter* G_*Anaerostipes* G_*Allobaculum* G_*Blautia*	↑	L	HFD/STZ mice	(70)
		G_*Lactobacillus* G_*Turicibacter* G_*Allobaculum*	↑	Sa	HFD/STZ mice	(70)
LDL cholesterol	Positive	G_*Romboutsia*	↓	L	HFD/GK rats	(66)
	Negative	G_*Prevotella*	↑	L	HFD/GK rats	(66)
Total cholesterol	Positive	S_*Prevotella* spp.	↓	M	OLETF rats	(50)
		S_*Clostridia* sp. S_*Clostridiales* spp. S_*Oscillospiraceae* sp. S_*Erysipelatoclostridium* sp. S_*Anaerotruncus* sp. *G3(2012)* S_*Firmicutes* sp. S_*Bacteroidales* sp.	↓	L	HFD mice	(65)
		G_*Romboutsia*	↓	L	HFD/GK rats	(66)
		S_*Clostridium cocleatum*	↑	M	HFD mice	(55)
	Negative	G_*Prevotella*	↑	L	HFD/GK rats	(66)
Triglyceride	Positive	S_*Prevotella* spp.	↓	M	OLETF rats	(50)
		G_*Romboutsia*	↓	L	HFD/GK rats	(66)
	Negative	G_*Prevotella*	↑	L	HFD/GK rats	(66)

*G, Genus; S, Species; M, metformin; Sa, saxagliptin; L, liraglutide; ↑, significantly increase; ↓, significantly decrease*.

## Discussion

Our study provides a comprehensive review to report human and animal data separately about reciprocal interactions between anti-hyperglycemic drugs and specific taxonomic groups of gut microbiota. While other reviews suggest the effects of anti-hyperglycemic drugs on gut microbiota without discerning findings from either human or animal studies (8, 11, 13), this systematic review attempts to fill the gap of these reviews to try to explore the associations among anti-hyperglycemic agents, specific taxonomic patterns of gut microbiota, and glucose control or metabolic profiles mainly in humans, as compared to those reported in mouse studies. Further, the fact that three-quarters of included studies were published in and after 2017 implies a growing interest in this clinical question for an up-to-date systematic review.

Of the 17 human studies selected, the majority of these studies focus on either newly diagnosed or prevalent T2D patients, and directed toward investigating the interplay of metformin, and to a lesser extent, acarbose, with gut microbiota. Our results suggest that these two drugs mediate their glucose-lowering effect, in part, by stimulating beneficial gut bacteria that could produce metabolites to promote intestinal homeostasis (3, 9). We rationalize that alterations in gut microbiota compositions might also underlie the gastrointestinal side effects known to metformin, i.e., diarrhea and fecal incontinence (10, 25, 80). In contrast, results from other anti-hyperglycemic drugs analyzed in this study showed inconsistency with respect to their effects on the compositions of gut microbiota, which might be attributable to small numbers of studies and, equally important, differences in animal models and experimental conditions used among these studies.

Further, treatment durations of anti-hyperglycemic drugs in available studies, regardless of human or animal, varied to a great extent (i.e., few days to few months). Thus, the reported drug effects on the gut microbiota structure were diverse. Indeed, the anti-hyperglycemic drugs, i.e., metformin, could affect the intestinal bacterial compositions after 1 or several days of treatment (24, 25, 40, 43), or after prolonged periods of treatments (21). For instance, Wu et al. found that gut microbiota compositions after a 2- and 4-month treatment of metformin in newly T2D patients were not identical (21). In contrast, Wang et al. did not find significant changes in the gut microbiota compositions among T2D patients after different periods of metformin or liraglutide treatment, given their baseline gut microbiota compositions were unknown (34). Thus, there are no consistent findings on gut microbiota after various treatment durations of anti-hyperglycemic drugs, and further studies are warranted to explore the treatment duration of anti-hyperglycemics required for emergence of beneficial gut bacteria.

Evidence indicates that the use of metformin or acarbose in T2D patients was associated with increases in the abundance of beneficial bacteria, including the genera *Bifidobacterium* (phylum *Actinobacteria*) and *Lactobacillus* (phylum *Firmicutes*), and the species *A. muciniphila* (phylum *Verrucomicrobia*). The increase in the genus *Bifidobacterium* was positively associated with diabetes control, which is consistent with that reported in the review by Gurung et al. (1). In addition, two included studies showed an increase in two specific species of the genus *Bifidobacterium* (*B. adolescentis* and *B. longum*), which was inversely associated with HbA1C levels or body weights, and positively associated with HDL cholesterol levels among newly diagnosed T2D patients (21, 32).

A number of human studies have reported positive associations between the abundance of the genus *Lactobacillus* (phylum *Firmicutes*) and improved T2D control (81, 82). For example, T2D patients treated with acarbose showed increased *L. gasseri* levels, accompanied by lower HbA1C and body weights (32). In addition, as several species in the genus *Lactobacillus* have been used as probiotics, administration of these *Lactobacillus* strains showed beneficial effects on glycemic control and lipid profiles in T2D patients (4). Moreover, almost all animal studies that tested the efficacy of several species from the genus as probiotics for T2D reported improvements of glucose parameters (1).

A previous report found decreased abundance in the mucin-degrading bacterium *A. municiphila* in patients with metabolic disorders, including obesity, impaired glucose tolerance, and diabetes, which were associated with insulin resistance, dyslipidemia, and overweight (83). Two other studies showed increased amounts of *A. municiphila* in newly diagnosed and prevalent T2D patients treated with metformin, which, however, did not provide pertinent information on metabolic parameters. The potential role of this mucin-degrading bacterium in ameliorating metabolic disorders was further confirmed by a series of animal experiments. For example, mice treated with metformin and liraglutide showed increased levels of *A. municiphila* in association with improved control of blood glucose and body weight (55, 65). More importantly, HFD-fed mice treated with *A. municiphila* exhibited similar improvements in glucose tolerance and goblet cell production and inflammatory regulations as compared to the metformin treatment group (56).

The effect of metformin and acarbose on the abundance of different species of the genus *Bacteroides* (phylum *Bacteroidetes*) is interesting. The genus *Bacteroides* seem to play a beneficial role in glucose metabolism where *B. intestinalis* and *B. vulgatus* were decreased in T2D patients, and *B. stercoris* was enriched in patients with diabetes remission (1). The same phenomenon was also noted in experimental animals (1). However, decreased abundance of some *Bacteroides* species, including *B. pleibeius, B. dorei, B. vulgatus*, after using acarbose in newly diagnosed T2D patients was reported to be positively associated with body weight in one study (32). As for rodent studies, colonization of *B. fragilis* was associated with more severe glucose intolerance in HFD-fed mice (24). A recent study, which compared fecal microbiota compositions between T2D patients and non-diabetic individuals, showed that *Bacteroides* was an independent risk factor of the disease by diminishing insulin sensitivity (84).

The effects of metformin on *A. muciniphila* were similar in both human and rodent studies. However, there was an inverse association of *Bacteroides* and metformin use in human and mouse studies in this review. Alterations of many other taxa in humans treated with metformin or acarbose were not the same as in mouse studies, and *vice versa*. The diverse dietary habits, metabolism or inflammatory statuses of host, body sizes and organs in these human and mouse studies might contribute to inconsistent findings of gut microbiota compositions (12, 85). Although the gut microbiota of human and mice are dominated by two major phyla, i.e., *Bacteroidetes* and *Firmicutes*, approximately 85% of the representative gut microbiota sequences in mice were not found in humans (86).

Further, the genera *Escherichia* and *Shigella*, belonging to the order of *Enterobacterales* in phylum *Proteobacteria*, were found to increase consistently after metformin treatment in T2D patients. Certain bacteria belonging to the phylum *Proteobacteria*, including the order *Enterobacteriales*, was found to be overly present in patients with metabolic disorders and T2D, and were positively related to intestinal permeability and endotoxemia in the pathophysiology of these metabolic diseases (87, 88). Enrichment in the order *Enterobacterales*, especially *Escherichia coli*, was demonstrated to play an important role in gut inflammation in patients with inflammatory bowel disease and also in various mouse model of colitis (88). Elbere et al. (25) observed an association between the severity of gastrointestinal side effects and increased abundance of the genera *Escherichia* and *Shigella*. Thus, the enrichment of the order *Enterobacteriales* might contribute to gastrointestinal side effects of metformin.

In this review, the results of β-diversity indicate significant changes in gut microbiome structure related to metformin or acarbose treatment. The findings on α-diversity among those treated with metformin were inconsistent while the richness and evenness were decreased after treating with acarbose. For healthy human subjects, the reference microbiome list and abundance profile showed various ratios of *Bacteroidetes* and *Firmicutes*, as well as the other phyla, e.g., *Acinobacteria, Proteobacteria* (89). This might reflect sufficient α- and β-diversities in healthy individuals due to significant regional heterogeneity at the species level and consistency at the higher taxonomic level (89). With regard to T2D patients, the associations between the disease and the diversities of microbiota were inconclusive (1). In the other words, there is no consensus or simple way to make a conclusion on the relationship between diversity and gut microbiota compositions among T2D patients treated with metformin or acarbose.

While metformin and acarbose have been shown to stimulate the growth of SCFA-producing bacteria, e.g., *Lactobacillus* and *Bifidobacterium*, information on the effects of these drugs on the fecal levels of various SCFAs in humans is lacking in the literature. Thus, this review entailed data from mouse studies, which are more informative. These mouse studies showed an increase trend in fecal and cecal levels of acetate, propionate, and butyrate in response to treatments of metformin, acarbose, voglibose, dapagliflozin or canagliflozin, of which the impacts on other physiological functions, other than that in gastrointestinal track, warrant further evaluations (90). With respect to bile acids, information on the effects of anti-hyperglycemic agents on their levels is limited and often conflicting in human vs. rodent studies, which merits further investigations to understand the role of other confounding factors, such as diets, antibiotic therapy, and disease states (91).

Again, the most critical limitation is lack of consistency among human and rodent studies. In humans, differences in the health status of participants, disease type or staging, ethnicity, drug dosage, and duration of treatment might directly impact gut microbiota compositions. Furthermore, it becomes difficult to come to a conclusion due to the small number of participants as well as differences in study design in each study. The risk of bias of studies needs to be taken into considerations, in which overall bias of randomized trials was high risk and unclear risk, and two out of five quasi-experimental studies and all of observational studies were at serious risk. Also, there was a high degree of heterogeneity in rodent studies due to differences in species used and environmental factors, as alluded above. Differences in microbiota analysis methods could also be a cause of deviation.

Another major limitation is lack of human studies on anti-hyperglycemic drugs beyond metformin and acarbose. Thus, no conclusion could be reached regarding the associations between human gut microbiota and these drugs. Because results from rodent models might not always be translatable to humans, conclusions should be made with cautions. Although findings from rodent studies included in this review suggest potential positive effects of other anti-hyperglycemic drugs besides metformin and acarbose on human gut microbiota, additional human studies on these drugs are needed to clarify the role of gut microbiota in their therapeutic efficacies.

In light of the enormous amounts of published data, this systematic review aims to provide readers a comprehensive view of this emerging area by taking an integrated approach through an all-inclusive literature search in conjunction with vigorous data extraction and validation, and assessment of risk bias. Moreover, this systematic review has tried to differentiate various aspects of the anti-hyperglycemic drug-gut microbiome-host axis, thereby filling the gap of merging all available data from human or animal studies relevant to the inter-dependence between anti-hyperglycemic drugs and the specific taxon of gut microbiota. Nevertheless, more investigations are warranted to support the positive contribution of metformin and acarbose to the health of gut microbiome (e.g., *A. muciniphila, Lactobacillus, Bifidobacterium longum*). In addition, given the limited information available in the literature, more studies are needed to shed light onto the roles of other anti-hyperglycemic drugs (e.g., miglitol, voglibose) in modulating human host taxa of gut microbiome.

## Conclusion

This review highlights that changes in specific taxa and β-diversity of gut microbiota were associated with metformin and acarbose in humans while pertinent information for other anti-hyperglycemic drugs could be only obtained in rodent studies. These results support the possible action mechanisms of these drugs, which may have translational potential to foster new approaches for the treatment of diseases related to gut dysbiosis in the future. Mouse studies on the other anti-hyperglycemics suggested the links between these drugs and gut microbiota were inconclusive. Therefore, additional human studies are needed to explore the role of gut microbiota in their therapeutic efficacies or side effects.

## Data Availability Statement

The original data presented in the study are included in the article/Supplementary Material, further inquiries can be directed to the corresponding author.

## Author Contributions

TC, P-CL, H-WL, CC, C-SC, K-CW, J-LH, and L-YY collected, screened, and extracted the data and analyzed the results. TC, H-WL, CC, and L-YY wrote the first draft of the manuscript. All authors contributed to conception, design of the study, manuscript revision, read, and approved the submitted version.

## Conflict of Interest

The authors declare that the research was conducted in the absence of any commercial or financial relationships that could be construed as a potential conflict of interest.

## Supplementary Material

Supplementary Figure 1 Risk of bias of randomized trials. **(A)** Each risk of bias item for each study. **(B)** Each risk of bias item across studies.

Supplementary Figure 2 Risk of bias of quasi-experimental studies. **(A)** Each risk of bias item for each study. **(B)** Each risk of bias item across studies.

Supplementary Figure 3 Risk of bias of observational studies. **(A)** Each risk of bias item for each study. **(B)** Each risk of bias item across studies.

Supplementary Figure 4 Risk of bias of animal studies. **(A)** Each risk of bias item for each study. **(B)** Each risk of bias item across studies.

Supplementary Table 1 Searching strategy.

Supplementary Table 2 Characteristics of housing and dietary of studied animals.

Supplementary Table 3 Effects of anti-hyperglycemia drugs on level of short-chain fatty acids (SCFAs).

Supplementary Table 4 Effects of anti-hyperglycemia on bile acid levels.
