# Supplementary figures and images for: Effects of Non-insulin Anti-hyperglycemic Agents on Gut Microbiota: A Systematic Review on Human and Animal Studies
Source: Front Endocrinol (Lausanne). 2020 Sep 23;11:573891. doi: 10.3389/fendo.2020.573891 (PMC7538596; doi:10.3389/fendo.2020.573891)

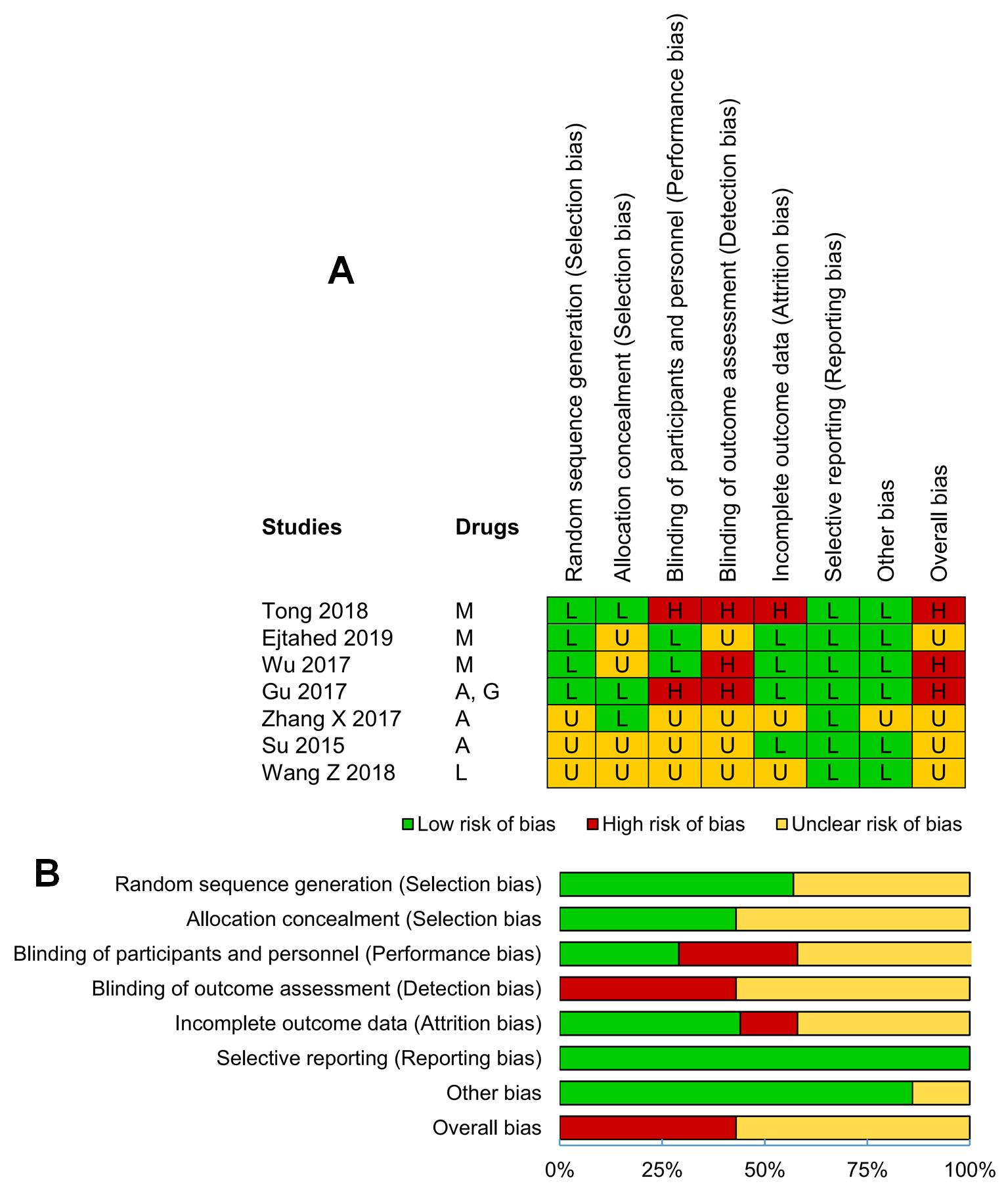

Supplement: Supplementary Figure 1 — Risk of bias of randomized trials. (A) Each risk of bias item for each study. (B) Each risk of bias item across studies. [file Image_1.JPEG]

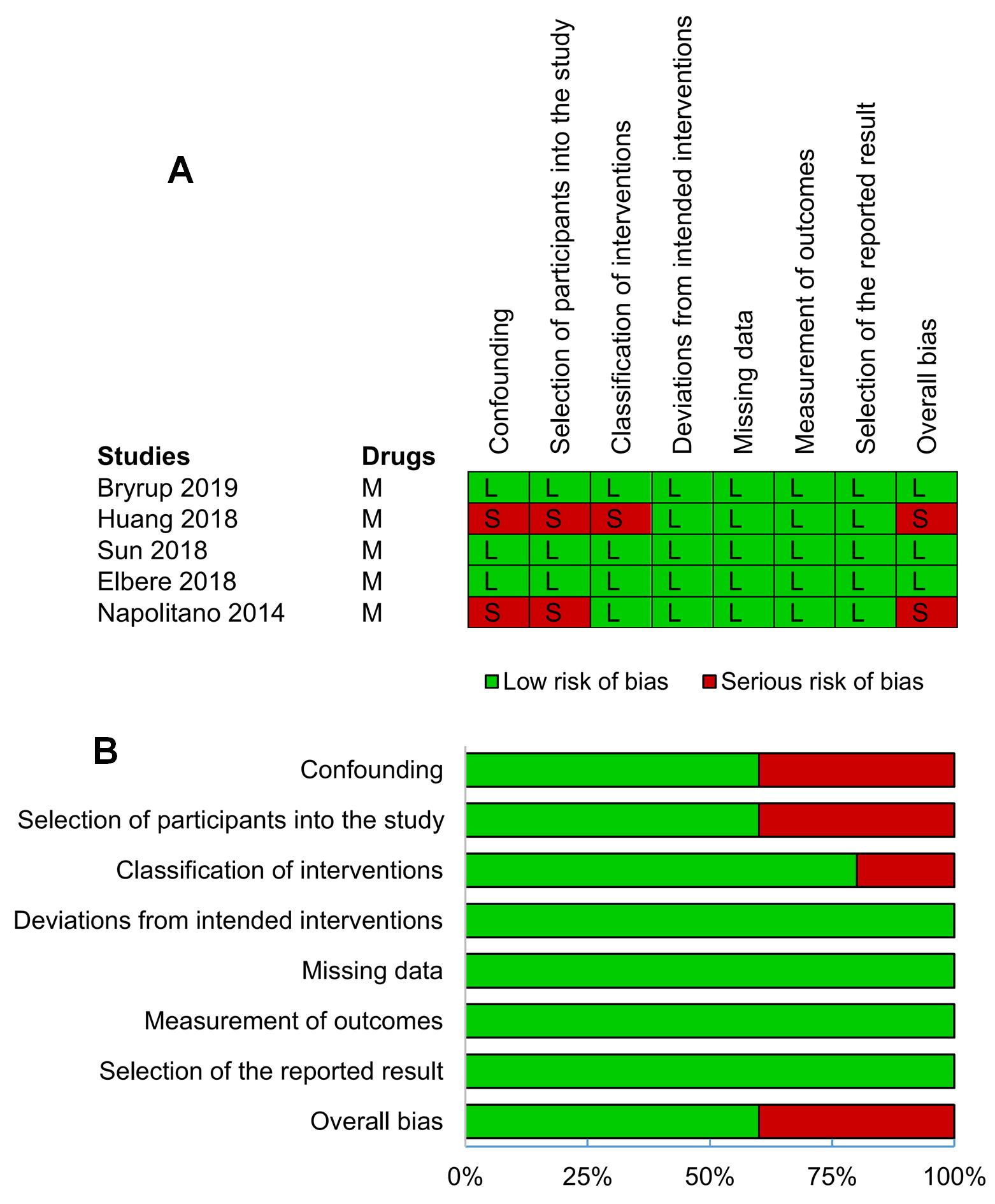

Supplement: Supplementary Figure 2 — Risk of bias of quasi-experimental studies. (A) Each risk of bias item for each study. (B) Each risk of bias item across studies. [file Image_2.JPEG]

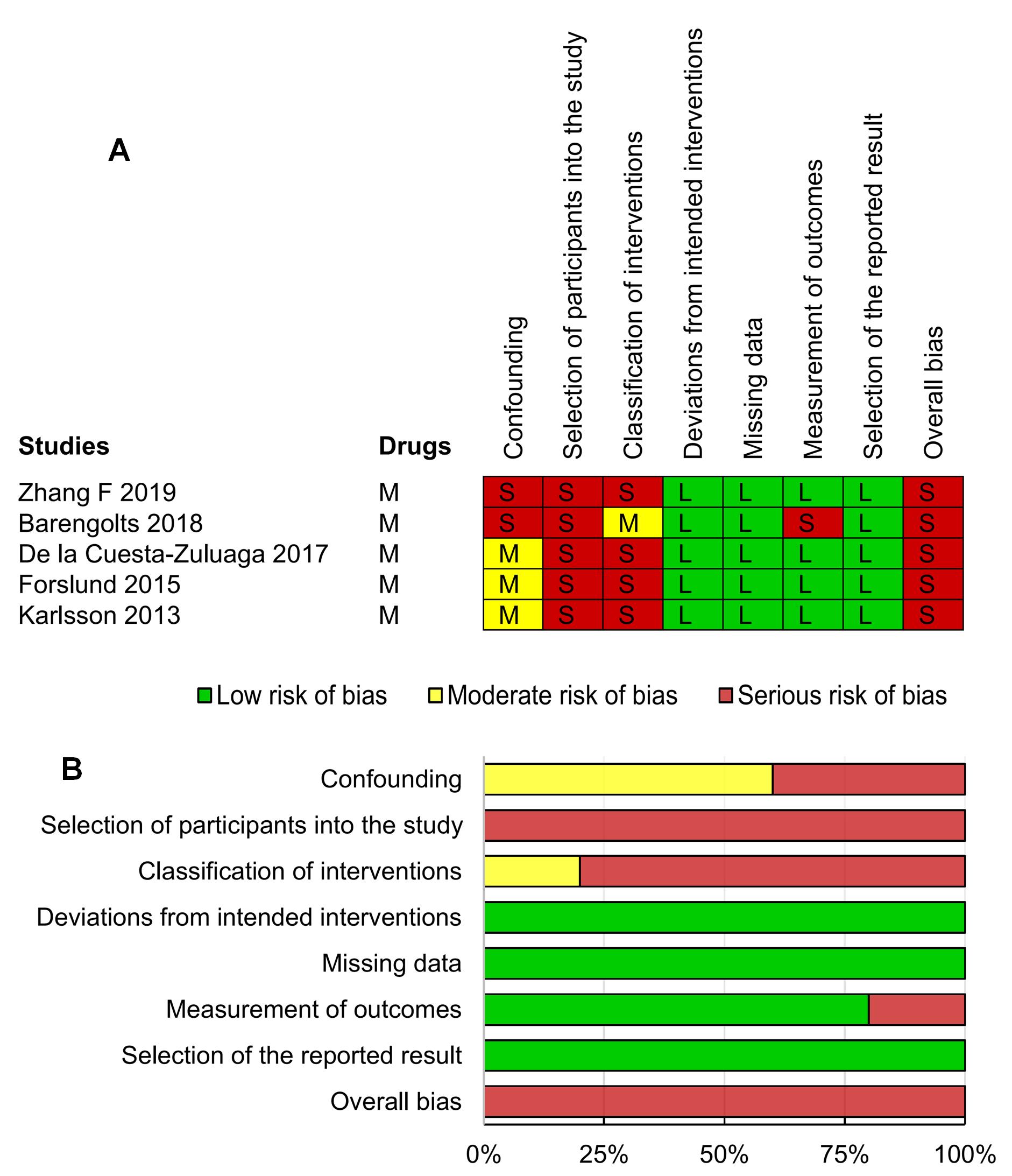

Supplement: Supplementary Figure 3 — Risk of bias of observational studies. (A) Each risk of bias item for each study. (B) Each risk of bias item across studies. [file Image_3.JPEG]

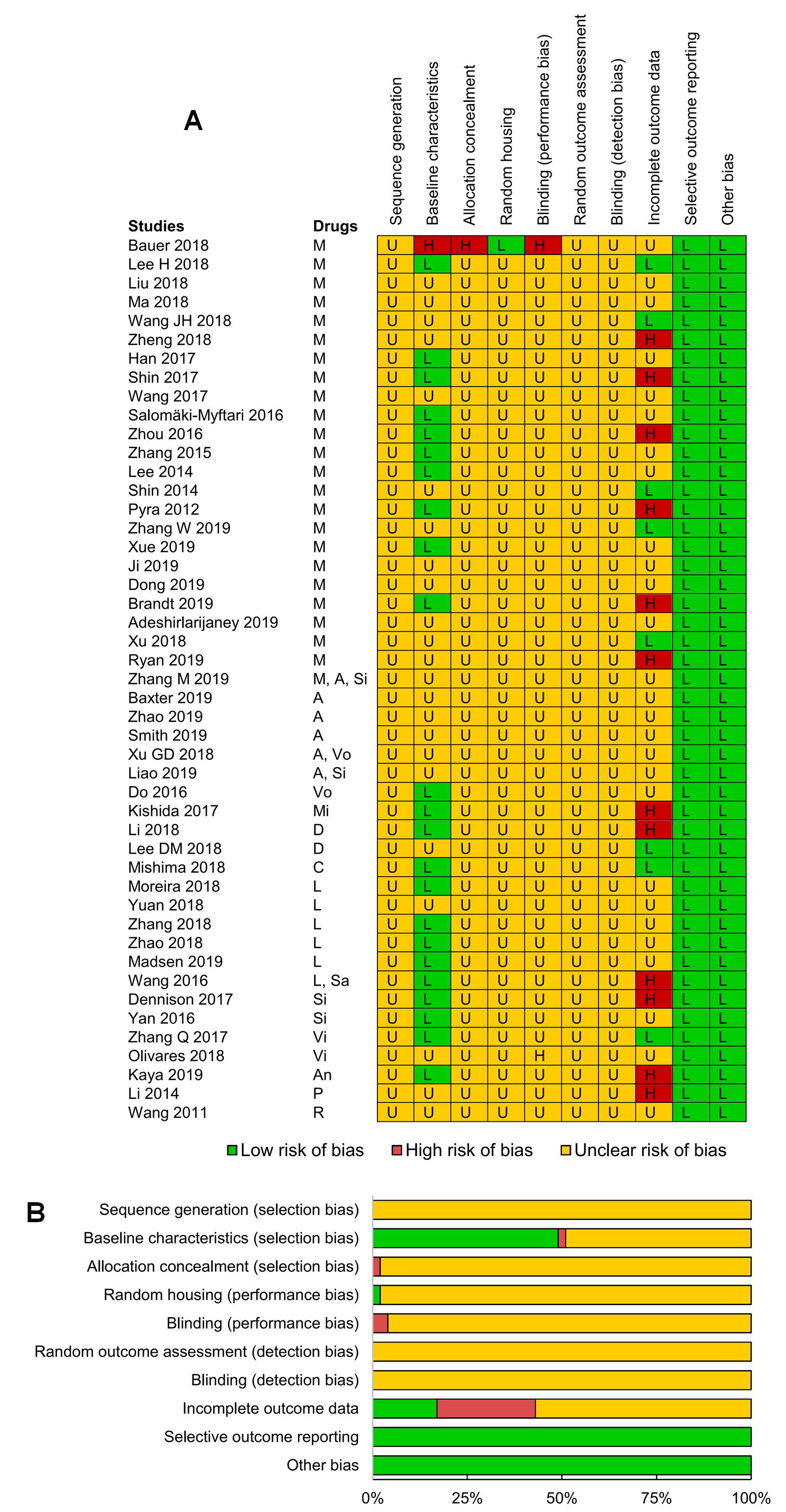

Supplement: Supplementary Figure 4 — Risk of bias of animal studies. (A) Each risk of bias item for each study. (B) Each risk of bias item across studies. [file Image_4.JPEG]
