# Supplementary material for: Effects of Non-insulin Anti-hyperglycemic Agents on Gut Microbiota: A Systematic Review on Human and Animal Studies
Source: Front Endocrinol (Lausanne). 2020 Sep 23;11:573891. doi: 10.3389/fendo.2020.573891 (PMC7538596; doi:10.3389/fendo.2020.573891)
Supplement: Supplementary Table 1 — Searching strategy. [file Table_1.DOCX]

**Supplementary Table 1.** Searching strategy

| **Database** | **PubMed** | **Embase** | **Scopus** |
| --- | --- | --- | --- |
| Search terms - 1 | flora[tiab], microbiome[tiab], microbiota[tiab], microflora[tiab], microbes[tiab], microbial[tiab], microorganisms[tiab] | flora, microbiome, microbiota, microbial, microbes, microorganisms | flora, microbiome, microbiota, microbial, microbes, microorganisms |
| Search terms - 2 | Antihyperglycemic*[tiab], Hypoglycemic*[tiab], Antidiabetic*[tiab], Glucose control[tiab], Glucose lower*[tiab], Biguanide*[tiab], Metformin[tiab], glibenclamide[tiab], chlorpropamide[tiab], tolbutamide[tiab], tolazamide[tiab], carbutamide[tiab], glipizide[tiab], gliquidone[tiab], gliclazide[tiab], metahexamide[tiab], glisoxepide[tiab], glyburide[tiab], glimepiride[tiab], acetohexamide[tiab], glymidine[tiab], Dipeptidyl Peptidase IV Inhibitor*[tiab], Dipeptidyl Peptidase 4 Inhibitor*[tiab], sitagliptin[tiab], vildagliptin[tiab], saxagliptin[tiab], alogliptin[tiab], linagliptin[tiab], gemigliptin[tiab], evogliptin[tiab], Sodium glucose cotransporter 2 inhibit*[tiab], SGLT2 inhibit*[tiab], dapagliflozin[tiab], canagliflozin[tiab], empagliflozin[tiab], ertugliflozin[tiab], ipragliflozin[tiab], alpha Glucosidase Inhibit*[tiab], acarbose[tiab], miglitol[tiab], voglibose[tiab], Thiazolidinedion*[tiab], pioglitazone[tiab], rosiglitazone[tiab], troglitazone[tiab], exenatide[tiab], liraglutide[tiab], lixisenatide[tiab], albiglutide[tiab], dulaglutide[tiab], semaglutide[tiab], nateglinide[tiab], repaglinide[tiab], mitiglinide[tiab], benfluorex[tiab] | Antihyperglycemic*, Hypoglycemic*, Antidiabetic*, Glucose control, Glucose lower*, Biguanide*, Metformin, glibenclamide, chlorpropamide, tolbutamide, tolazamide, carbutamide, glipizide, gliquidone, gliclazide, metahexamide, glisoxepide, glyburide, glimepiride, acetohexamide, glymidine, Dipeptidyl Peptidase IV Inhibitor*, Dipeptidyl Peptidase 4 Inhibitor*, sitagliptin, vildagliptin, saxagliptin, alogliptin, linagliptin, gemigliptin, evogliptin, Sodium glucose cotransporter 2 inhibit*, SGLT2 inhibit*, dapagliflozin, canagliflozin, empagliflozin, ertugliflozin, ipragliflozin, alpha Glucosidase Inhibit*, acarbose, miglitol, voglibose, Thiazolidinedion*, pioglitazone, rosiglitazone, troglitazone, exenatide, liraglutide, lixisenatide, albiglutide, dulaglutide, semaglutide, nateglinide, repaglinide, mitiglinide, benfluorex | Antihyperglycemic*, Hypoglycemic*, Antidiabetic*, "Glucose control*", "Glucose lower*", Biguanide*, Metformin, glibenclamide, chlorpropamide, tolbutamide, tolazamide, carbutamide, glipizide, gliquidone, gliclazide, metahexamide, glisoxepide, glyburide, glimepiride, acetohexamide, glymidine, "Dipeptidyl Peptidase IV Inhibitor*", "Dipeptidyl Peptidase 4 Inhibitor*", sitagliptin, vildagliptin, saxagliptin, alogliptin, linagliptin, gemigliptin, evogliptin, "Sodium glucose cotransporter 2 inhibit*", "SGLT2 inhibit*", dapagliflozin, canagliflozin, empagliflozin, ertugliflozin, ipragliflozin, "alpha Glucosidase Inhibit*", acarbose, miglitol, voglibose, Thiazolidinedion*, pioglitazone, rosiglitazone, troglitazone, exenatide, liraglutide, lixisenatide, albiglutide, dulaglutide, semaglutide, nateglinide, repaglinide, mitiglinide, benfluorex |
| Search fields | Tittle or abstract | Tittle or abstract | Tittle, abstract, or author keywords |

**Supplementary Table 2.** Characteristics of housing and dietary of studied animals

| Studies | Animal | Age | Housing before intervention | Acclimatization | Diet before intervention | Housing during intervention | Diet during intervention |
| --- | --- | --- | --- | --- | --- | --- | --- |
| Mice studies (n=30) | | | | | | | |
| Ryan 2019 | Male C57BL/6 | 3 w | Group-housed at 21^o^ C in a 12:12-h light/dark cycle | 1 w | HFD (60% kcal fat) for 12 weeks | Unchanged | Unchanged, for 12 weeks |
| Ji 2019 | Male C57BL/6J | 6 w | Group-housed, 12:12-h light/dark cycle, access to food pellets and water *ad libitum* | - | - | - | HFD (60% kcal fat) for 3 weeks |
| Adeshirlarijaney 2019 | Male C57BL/6 | 4-6 w | Group-housed | 2 w | Standard grain-based chow for acclimatization, then HFD for 8-12 weeks | Unchanged | Unchanged |
| Liao 2019 | Male C57BL/6 | 3-4 w | NA | 2 w | HFD (kcal/100 g: 60% fat, 20% protein, 20% carbohydrate) for 14 weeks | NA | Unchanged, for 4 weeks |
| Madsen 2019 | Male C57BL/6 | 4 w | Single-housed in a 12:12-h light/dark cycle at 22 ± 1 °C; 50 ± 10% relative humidity | 2 w | HFD (60% kcal fat, energy density 5.21 kcal/g) for 35 weeks | Unchanged | NA |
| Wang JH, 2018 | SPF male C57BL/6J | NA | NA | 1 w | HFD (60% kcal fat) for 4 weeks | NA | Unchanged, for 10 weeks |
| Lee H 2018 | Male C57BL/6N | 6 w | Housed in a 12:12-h light/dark cycle at 22±2^o^ C and 55±5% humidity; access to food and water *ad libitum* | - | HFD (45% kcal fat) for 39 weeks | Unchanged | Unchanged, for 16 weeks |
| Zhou 2016 | Male C57BL/6J | 8 w | Housed in a 12:12-h light/dark cycle at 22±2^o^ C, 50±5% humidity; access to food and water *ad libitum* | - | HFD (60% calories from fat) for 18 weeks | Unchanged | Unchanged, for 4 weeks |
| Do 2016 | Male C57BL/6J | 5 w | NA | 1 w | - | Housed at 18–24 °C, 50–60 % humidity and a 12:12-h light/dark cycle; free access to food and water | HFD (AIN-76 rodent diet  with addition of 200 g fat/kg and 1 % cholesterol) for 12 weeks |
| Lee 2014 | Female C57BL/6 | 6 w | NA | - | HFD (60% lipid) for 28 week; or ND (5% lipid) | NA | Unchanged, for 10 weeks |
| Shin 2014 | C57BL/6 | 4 w | Group-housed; free access to food and water | - | ND or HFD (kcal/100 g: 60% fat, 20% protein, 20% carbohydrate) for 8 weeks | Unchanged | Unchanged, 6 weeks |
| Dong 2019 | Offspring of KC | 1 m | - | - | - | Free access to diet | HFCD for 2 months |
| Brandt 2019 | Female C57BL/6J | 6-8 w | - | - | - | Housed in a SPF facility, controlled conditions, free access to water | FFCD (60E% carbohydrates, 25E% fat, 15E% protein with 50% wt/wt fructose and 0.16% wt/wt cholesterol) for 4 days |
| Baxter 2019 | Male C57BL/6 | 8-12 w | Reared in a SPF facility | - | Standard diet for 4-8 weeks; or PPD (23.5% protein, 40.9% carbohydrate, 4.5% fat) for 4-8 weeks | Unchanged | HSD (12.4% protein, 68.3% carbohydrate, 4.1% fat) for 4 weeks, or PPD for 2 weeks |
| Kishida 2017 | Male C57BL/6J | 5 w | Housed in temperature- and light-controlled rooms, free access to food and water | 1 w | NA | Unchanged | HFDSD (%kcal: 44.6% fat, 40.6% carbohydrate, 14.8% protein) for 12 weeks |
| Olivares 2018 | Male C57BL/6J | 9 w | Group-housed in a pathogen-free environment, a 12:12 h day/light cycle, free access to food and water; | 1 w | Standard diet (10% kJ fat) for acclimatization | Unchanged | Western diet (45% kJ fat, 17% kJ sucrose) from 3rd week to 8th week of intervention |
| Zheng 2018 | Male C57BL/6J | 3 w | Housed at 25 °C, humidity of 55 ± 5%, a 12:12 h light/darkness cycle, *ad libitum* access to food and water | 1 w | HFD (fat 60.0%, carbohydrate 20.6%, protein 19.4%) for 4 weeks then injected with STZ intraperitoneally for 7 days | Unchanged | NA |
| Wang 2016 | Male ApoE -/- | 10 w | Bred in a pathogen-free environment with a 12 h light/dark cycle, free access to food and water | 1 w | Single dose STZ intraperitoneal injection | Unchanged | HFD (16.6% fat, 1.3% cholesterol) for 8 weeks |
| Xue 2019 | Female C57BL/6J | 21 d | Housed at a temperature-controlled 25°C, in a 12 h light/dark cycle room | - | DHEA and 60% HFD (energy: 19.4% protein, 20.6% carbohydrate, 60% fat) for 20 days | Unchanged | Normal diet for 21 days |
| Moreira 2018 | Male C57BL/6J | 8 w | NA | - | HFD (% calories: 57.2% fat, 27% carbohydrate, 15.8% protein) for 8 weeks | NA | Unchanged, for 2 weeks |
|  | Female *ob/ob* | NA | NA | - | - | NA | Regular chow for 15 days |
| Ma 2018 | Healthy C57BL/6 | NA | NA | - | Chow diet until 8 weeks of age | NA | Unchanged, for 30 days |
| Xu GD 2018 | Male ICR | 6 w | Housed at temperature 24°C, humidity 40–70% and a 12:12 h light/dark cycle, free to access food and water. | 1 w | ND for acclimatization | Unchanged | Unchanged, for 2 weeks |
| Zhang W 2019 | BKSLeprdb (*db/db*) | 6 w | Maintained under controlled light conditions in a 12:12 h light-dark cycle, access to food and water *ad libitum* | 2 w | ND for acclimatization | Unchanged | Unchanged, for 11 weeks |
| Lee DM 2018 | C57BLKS/J-leprdb/leprdb (*db/db*) | 8 w | Group-housed in a temperature and humidity controlled environment in a 12:12 h light–dark cycle, free access to food and water | 2 w | ND (15% fat, 65% carbohydrate, 20% protein) for acclimatization | Unchanged | Unchanged, for 8 weeks |
| Li 2018 | Male ICR MafA-deficient | 8 w | Group-housed in a well ventilated room, a 12:12 light/dark environment, free access to water and food | - | - | Unchanged | ND for 6 weeks |
| Li 2014 | Female KKAy | 10 w | - | - | NA | Single-housed in SPF condition, free access to food and water | NA |
| Wang 2011 | KKAy | NA | NA | NA | ND for 4 weeks, then HFD for 8 weeks | NA | NA |
| Smith 2019 | Offsprings of female CByB6 mF1/J and male C3D2F1/J | NA | NA | NA | LabDiet 5LG6 after weaning until 8 months of age | Mice were transferred every 14 days to fresh, ventilated cages, water provided in bottles. | Unchanged, until 762-973 days of age |
| Salomäki-Myftari 2016 | OE-NPY | 10-12 w | Housed on a 12:12 h dark/light cycle, access to food and water *ad libitum* | NA | NA | Unchanged | NA |
| Mishima 2018 | Male C57BL/6 | 7 w | NA | NA | CE-2 diet containing 0.2% adenine for 5 weeks (induce renal failure) | NA | CE-2 diet for 2 weeks |
| Rat studies (n=17) | | | | | | | |
| Bauer 2018 | Male SD | 8 w | Single-housed on a standard light-dark cycle, *ad libitum* access to food and water | NA | HFD (44% carbohydrates, 22% protein, 31% fat, 3.9 kcal/g) for 3 days | Unchanged | NA |
| Zhang 2015 | Male W | 8-10 w | NA | 2 w | HFD (60% kcal fat) for 10 weeks | NA | Unchanged, for 8 weeks |
| Pyra 2012 | Male SD | 8 w | NA | - | HFHSD (g/100 g: casein 20.0, sucrose 49.9, soybean oil 10.0, lard 10.0, Alphacel 5.0, AIN-93M mineral mix 3.5, AIN-93 vitamin mix 1.0, DL-methionine 0.3, and choline bitartrate 0.25) for 6 weeks | NA | Unchanged, for 7 weeks |
| Dennison 2017 | Female SD | 10 w | NA | - | HFHSD (g/100 g: casein 20.0, sucrose 49.9, soybean oil 10.0, lard 10.0, cellulose 5.0, mineral mix 3.5, vitamin mix 1.0, dl-methionine 0.3, choline bitartrate 0.25, and t-butylhydroquinone 0.002) for 14 weeks | NA | Control-93G diet for 8 weeks |
| Liu 2018 | Male W | Adult | Housed at 21-23 °C, 42–47% humidity, SPF environment, a 12:12-h light/dark cycle, *ad libitum* access to food and water. | 1 w | HFD (10% lard, 20% sucrose, 2.5% cholesterol, 1% sodium cholate, and 66.5% pulverized standard rat pellet) for 4 weeks, and a single dose of STZ intravenously injection | Unchanged | HFD, for 4 weeks |
| Xu M 2018 | SD | NA | Housed at 20-25^o^C, 50-70 humidity | NA | HFHSD for 15 days and intraperitoneal injection of STZ | Unchanged | NA |
| Zhang 2018 | Male SD | 5 w | Maintained at 23–25^o^C with a 12:12-h light/dark cycle | NA | HFD (kcal %: 45% fat, 20% protein, 35% carbohydrate; 4.73 kcal/g) for 4 weeks then STZ intraperitoneal injection | Unchanged | HFD until the end of the study |
| Zhang Q 2017 | Male SD | 5 w | Caged at 24 ± 1^o^C with lights for 12 h per day | NA | HFD (kcal %: 45% fat, 20% protein, and 35% carbohydrate; 4.73 kcal/g) for 4weeks then STZ intraperitoneal injection | Unchanged | HFD for 12 weeks |
| Yan 2016 | Male SD | 4 w | NA | 1 w | With/without HFHCD (19.8 g fat, 44.6 g carbohydrate, and 22.3 g protein per 100 g, and 40 kcal% fat, 40 kcal% carbohydrate, and 20 kcal% protein by energy) for 4 weeks, then STZ intraperitoneal injection | NA | With/without HFHC diet for 12 weeks |
| Yuan 2018 | Male SD | NA | Housed under a 12:12 h light/dark cycle, 22 ± 2°C | NA | ND, then STZ intraperitoneal injection | Unchanged | ND until the end of the study |
| Zhang M 2019 | Male ZDF | 7 w | Maintained on a 12:12 h light/dark cycle, controlled temperature 20–23°C, humidity 40–60% | - | Purina 5008 diet (16.7% fat, 26.8% protein and 56.5% carbohydrate) for 4 weeks | Unchanged | NA |
| Shin 2017 | Male OLETF | 6 w | Maintained on a 12:12 h light/dark cycle at temperature 25◦C and humidity 50–60%, free access to food and water | 6 w | Chow diet for 6 weeks | Unchanged | NA |
| Wang 2017 | Male OLETF | 4 w | NA | 6 w | NA | NA | NA |
| Han 2017 | Male OLETF | 4 w | Housed in a SPF facility, temperature 20 ± 2˚C, humidity 40-60%, a 12:12h light-dark cycle | 6 w | ND (20% protein, 4.5% fat, 63% calories from carbohydrate) for 6 weeks | Unchanged | Unchanged, for 12 weeks |
| Zhao 2019 | Male GK | 8 w | Housed in a SPF facilty,  temperature 21-26^o^C, 40-50%, a 12:12 h light/dark cycle, *ad libitum* access to food and water. | 1 w | Standard diet in acclimation | Unchanged | Unchanged, for 8 weeks |
| Zhao 2018 | Male W and GK | 3 w | Bred in a pathogen-free environment, at 22 ±2°C, a 12:12-h light/dark cycle, free access to food and water. | 1 w | HFD (40% carbohydrate, 20% protein, 40% fat) for 8 weeks | Unchanged |  |
| Kaya 2019 | Male OLETF | 16 w | Housed in under controlled conditions (23 ± 3^o^C; humidity: 50% ± 20%; 10–15 air changes/h; illumination 12 h/d), *ad libitum* access to tap water | - | Intraperitoneal injection of porcrine serum for 8 weeks | Unchanged | NA |

HFD = high-fat diet; ND = normal-chow diet; HFHSD = high-fat high-sucrose diet; HFHCD = high-fat high-carbohydrate diet; DHEA = trans-dehydroandrosterone; HFCD = high-fat high-calories diet; FFCD = fat-, fructose- and cholesterol-rich diet; HSD = high-starch diet; PPD = plant plolysaccharide diet; WD = Western diet; STZ = streptozocin intraperitoneal injection; DHEA = trans-dehydroandrosterone; PS = pocrine serum intraperitoneal injection; IP = Intraperitoneal injection; SD rats = Sprague-Dawley rats; ZDF rats = Male Zucker diabetic fatty rats, induced by leptin receptor gene knockout; KC mice = LSL-KrasG12D/+ and p48-Cre+/- mice, induced by LSL-KRASG12D and Cre alleles knockout; OLETF rats= Otsuka Long-Evans Tokushima Fatty rats, induced by spontaneous CCK_1_ receptor knockout; OE-NPY mice = homozygous transgenic OE-NPY mice, induced by transgenic mice overexpressing Neuropeptide Y under dopamine–β-hydroxylase promoter; W rats = Wistar rats; GK rats = Goto-Kakizaki rats, induced by polygenic Wistar substrain; *ob/ob* mice = mice model induced by *Lep^ob^* gene knockout; ICR MafA-deficient mice = model induced by targeted disruption of the mafA gene in ICR mice; *db/db* mice = model induced by mutation in the leptin receptor gene in mice; KKAy mice = induced by transfer the yellow obese gene (A^y^) into KK mice; m = months; w = weeks; d = days; NA = not available; - = no information*.*

**Supplementary Table 3**. Effects of anti-hyperglycemia drugs on level of short-chain fatty acids (SCFAs)

| **Drugs** | **Participants** | **Specimens** | **Acetate** | **Butyrate** | **Propionate** | **Studies** |
| --- | --- | --- | --- | --- | --- | --- |
| **Human studies** | | | | | | |
| Metformin | Obese woman | Feces | ↓ | ns | ns | Ejtahed 2019 |
|  | Newly T2D | Feces | ns | ↑^a^ | ↑^a^ | Wu 2017 |
|  | Prevalent T2D | Serum | ns | ns | ns | Huang 2018 |
| **Mouse studies** | | | | | | |
| Metformin | *db/db* mice | Feces | ↑ | ↑ | ns | Zhang W 2019 |
|  | Male OLETF rats | Feces | ↑ | ns | ns | Wang 2017 |
| Acarbose | ND mice | Feces | ↑ | ↑ | ↑ | Smith 2019 |
|  | ND mice | Ceca | ns | ↑ | ns | Xu GD 2018 |
|  | HSD mice | Feces | ns | ↑ | ns | Baxter 2019 |
|  | PPD mice | Feces | ↑ | ↑ | ns | Baxter 2019 |
| Voglibose | ND mice | Ceca | ↑ | ↑ | ↑ | Xu GD 2018 |
| Dapagliflozin | MafA-deficient mice | Feces | ↑ | ↑ | ↑ | Li 2018 |
| Canagliflozin | Renal failure mice | Ceca | ↑ | ↑ | ↑ | Mishima 2018 |
| Sitagliptin | HFD mice | Feces | ns | ns | ns | Liao 2019 |
| Vildagliptin | WD mice | Ceca | ns | ns | ↑ | Olivares 2018 |

↑ = significant increase; ↓ = significant decrease; ns = no significant difference; ^a^increase in men.

**Supplementary Table 4.** Effects of anti-hyperglycemia on bile acid levels

| **Drugs** | **Participants** | **Specimen** | **Total BAs** | **Primary BAs** | **Secondary BAs** | **Studies** |
| --- | --- | --- | --- | --- | --- | --- |
| **Human studies** | | | | | | |
| Metformin | Newly T2D | Feces and serum | ns | - | ↑^a^ | Sun 2018 |
|  | Prevalent T2D | Feces | ns | ns | ns | Wu 2017 |
|  |  | Feces | ns | ns | ns | Napolitano 2014 |
|  |  | Plasma | ↑ | ↑ | ↑ | Wu 2017 |
|  |  | Serum | ↓ | ↓ | ↓ | Napolitano 2014 |
| Acarbose | Newly T2D | Plasma | - | ↑^b^ | ↓^c^ | Gu 2017 |
|  |  | Feces | ↓ | ↑^d^ | ↓ | Gu 2017 |
| Glipizide | Newly T2D | Plasma | ns | ns | ns | Gu 2017 |
| **Mouse studies** | | | | | | |
| Metformin | OLETF rats | Liver tissue | ↓ | - | - | Han 2017 |
|  |  | Feces | ↑ | - | - | Han 2017 |
| Voglibose | HFD mice | Serum | - | ↑^e^ | ↓^f^ | Do 2016 |

BA = bile acid; ^a^increases in conjugated bile acids, including glycoursodeoxycholic acid (GUDCA) and tauroursodeoxycholic acid (TUDCA); ^b^increases in unconjugated bile acids, mainly cholic acids (CAs) and chenodeoxycholic acids (CDCAs); ^c^decreases mainly in conjugated deoxycholic acids (DCAs); ^d^increases in cholic acids (CAs) and chenodeoxycholic acids (CDCAs); ^e^increases in taurocholic acids (TCAs), cholic acids (CAs); ^f^increases in deoxycholic acids (DCAs); ↑ = significant increase; ↓ = significant decrease; ns = no significant difference; - = no information.
